# Genomic Characterization of Four Novel Probiotic Strains with Enzymatic Activity and Their Effects on Carp (*Cyprinus carpio*)

**DOI:** 10.3390/ani15131998

**Published:** 2025-07-07

**Authors:** Evgeniya Valeryevna Prazdnova, Maria Sergeevna Mazanko, Victoria Nikolaevna Shevchenko, Radomir Viktorovich Skripnichenko, Maksim Pavlovich Kulikov, Lilia Sergeevna Golovko, Vadim Alexeevich Grigoriev, Tatiana Alexandrovna Maltseva, Daria Borisovna Kulikova, Dmitry Vladimirovich Rudoy

**Affiliations:** Research Laboratory «Agrobiotechnology Center», Don State Technical University, Gagarina Sq. 1, Rostov-on-Don 344002, Russiavshevchenko@donstu.ru (V.N.S.); aqua-group@yandex.ru (V.A.G.); tamalceva@donstu.ru (T.A.M.); rudoy.d@gs.donstu.ru (D.V.R.)

**Keywords:** probiotic, *Bacillus*, aquaculture, lytic enzymes, non-ribosomal peptides, microbiota, feed conversion ratio

## Abstract

The shift toward plant-based feeds in aquaculture necessitates a search for probiotics to improve the digestibility of such feeds and fish growth. This study characterizes four *Bacillus* strains for their probiotic potential in carp (*Cyprinus carpio*). Strains isolated from river sediments underwent in vitro screening for proteolytic/amylolytic activity, antibiotic sensitivity, and biofilm formation. Whole-genome sequencing was performed and functional annotation identified probiotic-related genes. All strains were identified as *Bacillus velezensis* and harbored genes providing thermal, osmotic, and oxidative stress resistance, alongside enzymes and bioactive metabolite synthesis pathways. Two non-antagonistic strain pairs were solid-phase cultivated on soybeans and incorporated into carp feed (0.1% *w*/*w*) for a 76-day trial, and growth metrics and gene expression were analyzed in juvenile fish. Group 1 (strains MT14 + MT42) exhibited a 40.75% higher biomass increase than the control group while Group 2 (strains MT141 + MT142) exhibited a 56.62% higher biomass growth. No significant gene expression changes occurred in juveniles, though MT141 + MT142 transiently modulated fry expression profiles. Therefore, these *Bacillus velezensis* strains synergistically enhance growth in carp that are fed plant-rich diets, highlighting their viability as aquaculture probiotics.

## Simple Summary

This study evaluates four novel *Bacillus* strains as probiotics to enhance plant-based feed utilization in carp aquaculture. Two preparations based on two pairs of synergistic strains significantly improved the weight gain of carp when added to feed on early stages of fish life, and genome analysis confirmed that these bacteria have the necessary traits to survive in the fish gut, such as resistance to gastrointestinal enzymes and acidic medium; to help digest food, while they have enzymatic activity that promotes the splitting of complex plant components; and fight harmful microbes by producing antimicrobial substances. No significant gene expression changes occurred in juveniles, though one of two preparations transiently modulated fry expression profiles. The results indicate that the mechanism of action of these probiotics is primarily mediated by their enzymatic activity. Beyond characterizing novel aquaculture probiotics, this work identifies genetic markers for the selection of potential probiotic strains in the future.

## 1. Introduction

Aquaculture is a rapidly developing area playing a highly important role in the provision of food security, especially in developing countries. It provides people with an accessible protein source and ensures the economic growth of coastal and rural regions [1,2]. The scarcity of fish in natural water bodies caused by overfishing and pollution further emphasizes the need to develop intensive and sustainable fish farming [3]. Demand for seafood is expected to double by 2050, and aquaculture has a crucial role to play in meeting this demand [4].

Intensive fish farming comes with certain challenges, such as high stress levels in farmed fish [3]. The economic side of commercial aquaculture is also an important issue. A significant part of the costs in intensive fish farming (40–70%) is spent on feed [5]. In carp farming, feed costs have increased by 60–80% in recent years [6].

Another challenge is growth stimulation. Rapid individual weight gain in fish is known to improve winter survival under conditions of boreal and continental climates when water temperatures drop to critical values and hydrobionts’ life preservation depends solely on reserves accumulated during the growing season. In European carp farming, there are two wintering periods before the fish reach commercial size [7].

For a long time, the main source of protein in feed has been fishmeal, but its limitations force us to look for alternatives. Therefore, the use of vegetable ingredients (soy, rapeseed, and various grains) is increasing: [8,9,10]. Fish are unable to produce cellulolytic enzymes, and the presence of cellulose in feed can be a problem affecting the digestibility of food. Adding probiotic bacteria to food that produces cellulolytic exoenzymes can improve the digestibility and assimilation of feed with plant components in the composition [11,12,13]. The prevention of pathological conditions by functional feeds is a preferable strategy for the aquaculture industry than treating existing diseases [14]. Emphasis is now being placed on developing more environmentally friendly and sustainable methods of disease management, such as the use of probiotic microorganisms, included in feed formulations [15].

Probiotics are live microorganisms that, when administered in sufficient quantities, have beneficial effects on the host organism’s health [16]. In aquaculture, their application shows promising results, improving various aspects of fish health and ecosystem stability. Probiotic microorganisms stimulate the immune system, increasing the resistance of macroorganisms to infectious diseases. Additionally, they improve digestion, facilitating more efficient nutrient absorption from feed and resulting in enhanced growth rates and feed conversion ratios [17,18].

Bacteria of the genus *Bacillus* rank among the most commonly used probiotics in aquaculture [19]. One of the most important features of *Bacillus* is its ability to form endospores. These spores exhibit remarkable resilience to adverse environmental conditions such as heat, drying, and exposure to disinfectants. Their durability ensures long-term bacterial viability, which is an important factor for aquaculture applications [15].

*Bacillus* species are widespread in nature and can be found in soil [20], water [21], and fish intestines [22]. Strains used in aquaculture are isolated from various sources: soil, water from fish farms, fish intestines, and commercial preparations [20,21,23,24,25,26]. Notably, many *Bacillus* species inhabiting the intestines of fish are autochthonous microorganisms, i.e., they are natural inhabitants of this environment and form part of the normal microflora [3,27]. *Bacillus* strains are suitable probiotics for aquaculture because they are frequently found in both freshwater and seawater and the gastrointestinal tract of animals [28]. Some *Bacillus* probiotics were isolated from the gastrointestinal tract of healthy fish [29,30,31,32,33,34]. This indicates that they are well adapted and tend to colonize rather than just transit through the gastrointestinal tract [35], underscoring the safety and efficacy of *Bacillus*-based probiotics in aquaculture [3].

Probiotics containing enzymes such as protease, amylase, cellulase, and xylanase can significantly improve feed efficiency and growth performance in various aquaculture species such as white shrimp (*Litopenaeus vannamei*), zebrafish (*Danio rerio*), common pangasius (*Pangasius bocourti*), European trachinotus (*Trachinotus ovatus*), and Nile tilapia (*Oreochromis niloticus*) [30,36,37,38,39,40]. Various studies indicate that *Bacillus* can produce digestive enzymes such as amylase, protease, lipase, lipase, cellulase, and xylanase [35,41,42]. Studies demonstrate that enzymes secreted by bacteria of the genus *Bacillus* enhance nutrient assimilation in marine animals from cost-effective plant-based resources [43]. The introduction of probiotic strains, in addition to realizing antagonistic and immunomodulatory effects, also improves nutrient uptake efficiency, including in the larval stages of ontogenesis. Probiotic strains can improve activity of digestive enzymes such as lipase, protease, and amylase, leading to better growth and survival of fish [44,45].

Bacilli are recognized as natural inhabitants of the carp gut microbiota [46]. Therefore, bacilli-based probiotics are more likely to successfully colonize and exert beneficial effects in fish than species uncharacteristic of carp. Furthermore, bacilli are able to form spores that survive drying, which greatly facilitates the production, storage, and industrial application of probiotic formulations [47]. For these reasons, bacilli were selected as the focus of our search for new probiotics.

Thus, probiotics with an enzymatic activity contribute to more efficient feed digestibility, increasing the growth rate in commercial aquaculture facilities [12]. The objective of this work is to isolate new probiotic strains with enzymatic activity for aquaculture, characterize their properties through microbiological assays and full-genome sequencing, and evaluate their effects on carp.

## 2. Materials and Methods

### 2.1. Strain Isolation and Growth

Bottom sediments were collected from the Don River, at the experimental research base of the Southern Scientific Center of the Russian Academy of Sciences, Kagalnik, Rostov Region, Russia (47.09619 north latitude, 39.30526 east longitude). Samples were taken at the depth 0–5 cm, pasteurized (95 °C, 5 min) and immediately inoculated on test media.

The bacteria were incubated for 24 h at 30 °C. Proteolytic activity was tested on solid nutrient LB (Luria–Bertani) medium supplemented with 200 mL/liter milk (Groupe Danone, Krasnodar, Russia). Colonies surrounded by clear zones (casein hydrolysis) were selected. Amylolytic activity was tested on LB agar with the addition of 10 g/L starch (SP-Don, Rostov-on-Don, Russia). Colonies with clear zones (starch degradation) after iodine staining were selected. Cellulolytic activity was tested on solid LB supplemented with 10 g/L carboxymethylcellulose (LenReactiv, St. Petersburg, Russia). After Congo red (LenReactiv, St. Petersburg, Russia) staining, colonies with unstained zones (CMC hydrolysis) were selected. Inoculation was carried out in 20 replications on each medium.

During isolation of probiotic *Bacillus* from natural sources and intestinal contents, colony morphology on solid media was the primary selection criterion. *Bacillus* usually exhibit considerable morphological diversity, with colonies on the same nutrient medium varying in size, edge shape, colony height, color, density, surface texture, presence of wrinkles, their number, thickness, height, and location [48,49,50]. To maximize genetic diversity, morphologically identical strains from a single sample were excluded from further analysis.

Colonies that showed a high degree of activity (≥8 mm) were streaked onto LB agar and examined for strain purity using a depleting stroke. Purified strains were re-tested on respective media (4 colonies/plate), and clear zone diameters were measured. Reference strains *B. amyloliquefaciens* B1895 (GenBank number JMEG00000000) and *B. subtilis* KATMIRA, which have repeatedly demonstrated their probiotic activity in our previous studies, were used as controls [51,52,53].

### 2.2. Assessment of Strain Safety

To evaluate the safety of using the strains as probiotics, we investigated their antibiotic resistance as well as hemolytic activity.

Standard antibiotic disks were used for antibiotic resistance study: azithromycin 15 µg; amoxicillin/clavulanate 20/10 µg; ampicillin 10 µg; tetracycline 30 µg; gentamicin 10 µg; oxacillin 10 µg; clarithromycin 15 µg; cefazolin 30 µg; and ceftriaxone 30 µg (NICF, Tver, Russia). The tested strains were lawn-inoculated on Muller–Hinton agar (LenReactiv, St. Petersburg, Russia), then antibiotic disks were placed three per plate. Incubation was done for one day at 37 °C. After a day, inhibition zones were measured. Only strains with inhibition zones > 8 mm for any clinically important antibiotic were retained. Each test was carried out in three replications.

To assess hemolytic activity, selected strains were streaked onto blood agar (7% bull blood) and incubated for one day at 37 °C. Levels of β-hemolysis (complete lysis) or α-hemolysis (partial lysis) were recorded by color and width of halo. Non-hemolytic strains were selected for further work.

### 2.3. Strain Compatibility Testing

On LB agar plates, two strains of potential probiotic bacilli were streaked in parallel lines on undried dishes 5 mm apart. All pairwise combinations of strains were tested. Plates were incubated for 48 h at 37 °C and then interaction zones were analyzed. Each test was carried out in two replications.

### 2.4. Probiotic Preparation Using Solid-Phase Fermentation

Preparations were produced based on *B. velezensis* MT14, *B. velezensis* MT42, *B. velezensis* MT141, and *B. velezensis* MT142 strains, each strain prepared separately. Petri dishes containing LB medium were inoculated with the corresponding probiotic strain and incubated for 48 h at 37 °C. The resulting plates served as the starting material for the preparation of the biopreparation. Soybeans were taken on the basis that 1.2 kg of beans is required to obtain 1 kg of the final probiotic product. Soybeans were washed and soaked for 12 h at 20–22 °C. Then the hydrated soybeans were sterilized in an autoclave at 121 °C for 40 min in a food-grade aluminum container.

After cooling to 60 °C (controlled by a mercury thermometer disinfected with 70% ethanol), the starters were added to the containers and mixed thoroughly with a sterile food-grade stainless steel spoon, then incubated for 48 h at 40 °C. During this time, the biofilm of bacilli grew on a soybean.

After incubation, the substrate was mixed using a sterile spoon and ground on a pre-sterilized hand grinder. The resulting mass was placed on food-grade plastic pallets sterilized with 70% ethyl alcohol and 3% hydrogen peroxide. The preparation was carefully leveled with a layer of about 1 cm and dried on thermal mats at 40–45 °C for four days. Then the mass was turned over and dried for another three to five days until drying was complete. The preparation was ground on a ML-50B mill (Biobase, Jinan, China), the bowl of which was pre-treated with 70% ethyl alcohol and 3% hydrogen peroxide.

The bacterial count in the preparation was determined by grinding it with water (1:10) and preparing a series of decimal dilutions, which were inoculated superficially onto solid nutrient medium LB. The resulting powders were mixed in pairs to achieve the same amount of each strain in the preparation. The final probiotic spores count in the preparations was 3.7·10^8^ CFU/g for MT42 + MT14, and 6.4·10^8^ CFU/g for MT141 + MT142.

### 2.5. Experimental Diet

The diet of individuals included specialized starter mixed feeds for young carp with a pellet size of 2 mm. Production of experimental feeds consisted of the following stages: weighing of components in accordance with the recipe (laboratory scales ADAM HCB 1002, Dongguan, China, were used for weighing), mixing of dry components of feeds (including the probiotic supplement and moistening to a mass fraction of moisture 12% (mixing was carried out in a horizontal SG-1.5 mixer, (manufacturer AgroPostavka LLC, Nizhny Novgorod, Russia), water was added to the mixer through nozzles mounted on the mixer body), pelleting (carried out on a ZLSP-150 compound feed granulator with a 2 mm matrix installed, manufactured by AgroPostavka LLC, Nizhny Novgorod, Russia), cooling and sieving of pellets (carried out in a cooling column and sieving of KO 5.5 granules, manufactured by AgroPostavka LLC, Nizhny Novgorod, Russia), and oiling of pellets under vacuum (carried out in a vacuum granule oiler, manufactured by IE Shelkunov, Krasnodar Krai, Russia). Feed for the experiment was made from the following components: fish meal (27%) (Livadia-N LLC, Belgorod Region, Russia); wheat flour (17.24%) (Yug Russi LLC, Rostov-on-Don, Russia); chicken meat and bone meal (12.5%) (Livadia-N LLC, Belgorod Region, Russia); soybean meal (10%) (ROSAGROCORM LLC, Novosibirsk, Russia); pork meat meal (8%) (Livadia-N LLC, Belgorod Region, Russia); blood meal (6%) (Trading House “Feed Flour Factory” LLC, Republic of Tatarstan, Russia); corn meal (5%) (ROSAGROCORM LLC, Novosibirsk, Russia); corn gluten (3%) (ROSAGROCORM LLC, Novosibirsk, Russia); carp fish premix (4%) (manufacturer VetAgroSnab LLC, Rostov-on-Don, Russia); linseed oil (3%) (Len OK LLC, Nizhny Novgorod Region, Russia); fish oil (3%) (manufacturer NVC Agrovetzashita LLC, Moscow, Russia); table salt (NaCl) (1%) (manufacturer Russol LLC, Orenburg, Russia); calcium lignosulfonate (pellet fixer) (0.1%) (manufacturer Polyplast JSC, Russia); probiotic preparation (0.1%) (produced in the Russia, Rostov-on-Don, DSTU); and astaxanthin (0.06%) (manufacturer VetAgroSnab LLC, Rostov-on-Don, Russia). Two probiotic preparations were used: the first consisted of a mixture of probiotic strains *Bacillus velezensis* MT14 and *B. velezensis* MT42 (the same concentration), the second consisted of a mixture of probiotic strains *B. velezensis* MT141 and *B. velezensis* MT142 (the same concentration). The nutritional composition of the feed was developed taking into account the following target indicators: crude protein content—approximately 47% (±1.5%), crude fat content—approximately 13% (±1.5%), crude ash content—approximately 5% (±0.6%), and crude fiber content—approximately 2% (±0.5%). The water resistance of the granule was 35 min (±0.5 min).

The daily feeding rate was 3% of the fish biomass in the pool. Feeding was carried out every 4 h daily. Probiotic strains *Bacillus velezensis* MT14 and *Bacillus velezensis* MT42 were included in the diet of experimental group No. 1. *Bacillus velezensis* MT141 and *Bacillus velezensis* MT142 were included as part of the diet of experimental group No. 2. The content of probiotic preparation in the total composition of feed components was 0.1%. The diet of the control group included feed without any probiotic added. To confirm the persistence of bacilli in the feed, the feed was rubbed with water (1:10) and a series of decimal dilutions were prepared and inoculated superficially onto LB solid nutrient medium. The dishes were incubated for 24 h at 37 °C and then counted.

### 2.6. Experimental Design and Fish Conditions

The study was approved by the Local Independent Ethical Committee of the Don State Technical University (Rostov-on-Don, Russia). During the experiments, the recommendations for humane and ethical treatment of laboratory animals were followed, including in accordance with Directive 2010/63/EU of the European Parliament and of the Council of 22 September 2010 “On the Protection of Animals Used for Scientific Purposes”.

Juvenile carp *Cyprinus carpio* Linnaeus, 1758 in the amount of 450 specimens obtained in July 2024 from producers kept in the pond fish farm (Rostov Region, Russia) were used for the study. At the start of experiment the fish were clinically healthy, with no external signs of diseases. Health status (excess mucous secretion, normal coloration, erosion of scales or fins, skin, exophthalmos and presence of cysts, spots, or areas on the body and gills) and behavioral signs (swimming and feeding reflexes) were controlled by physical examination [54]. Fish were randomly distributed into three experimental groups: control (150 specimens), experiment No. 1 (150 specimens) and experiment No. 2 (150 specimens). The duration of the experiment was 76 days.

The fish were kept in round tanks as part of a recirculating aquaculture system (RAS), which included mechanical and biological filtration of recycled water, ultraviolet radiation treatment for disinfection, and an oxygenator for aerating purified water. Each tank was made of polypropylene and had a diameter of 150 cm and height of the water column of 70 cm. At the beginning of the experiment, 150 juvenile carp were placed in each tank (121 specimens/m^3^). Mortalities were recorded daily. The biomass of fish at the beginning of the experiment was 0.57 kg in the control group, 0.55 kg in experimental group No. 1, and 0.44 kg in experimental group No. 2.

During the experiment, the water temperature in the experimental pools averaged 21.95 ± 0.19/(21.58–22.37)°C [55]. The concentration of oxygen (O_2_) dissolved in water during the experiment stayed within the optimal range for juvenile carp and averaged 8.98 ± 0.21(8.53–9.46) mg/L. The pH of the water was on average 7.62 ± 0.45/(7.49–8.01), which are normal values for carp content [56].

To minimize handling, control measurements of the size and mass values of fish were carried out twice during the experiment period: at the beginning and at the end. At the end of the experiment, feeding was stopped for 24 h. A random sample of 60 specimens was taken from each group, and they were anesthetized using clove powder (150 mg/L) [57] to determine biometric parameters. The length of the fish was measured to the end of the scale cover (L, cm) using a Gigant 0–300 mm GSMI-300 digital vernier caliper (LLC Gigant-Pribor, Izhevsk, Russia) with a measurement accuracy of 0.01 mm. The mass of fish (m, g) was determined using laboratory scales of accuracy class II BEL LG-2202i with a discreteness of 0.01 g (ChangZhou XingYun Electronic Equipment Co., Changzhou, China).

To evaluate efficiency of feeding, calculation of the following parameters was made [58,59,60,61,62]

- Fulton’s fatness coefficient (QF, conventional units) according to the following formula:

$$Q_{F}=\frac{m*100}{l^{3}}$$

- individual biomass growth (WGi, g) using the following formula:

$$WGi=m_{1}-m_{0}$$

- total biomass growth (WGt, kg) according to the following formula:

$$WGt=M_{1}-M_{0}$$

- feed conversion rate (FCR, kg/kg) basing on the following formula:

$$FCR=\frac{M_{f}}{WGt}$$

- fish survival rate (S, %) estimated with the following formula:

$$\;S=100*\;\frac{N_{1}}{N_{0}}$$

- specific rate of weight growth (SGRW, %/day) determined according to the following formula:

$$\;SGRW=100*\;\frac{In\left(m_{1}\right)-In\left(m_{0}\right)\;}{t}$$

- specific growth rate of fish in length (SGRL, %/day) using the following formula:
  $$SGRL=100*\;\frac{In\left(l_{1}\right)-In\left(l_{0}\right)\;}{t}$$
  where t—duration of the experiment, days; l_1_—length at the end of the experiment, cm; l_0_—length at the beginning of the experiment, cm; m_1_—weight at the end of the experiment, g; m_0_—weight at the beginning of the experiment, g; N_1_—number of fish at the end of the experiment, specimens; N_0_—the number of fish at the beginning of the experiment, specimens; M_f_—feed weight, kg; M_1_—feed weight, kg; M_0_—fish biomass at the beginning of the experiment, kg; m—weight, g; l—length, cm.

The significance of the differences in the obtained values was determined using Student’s parametric criterion.

### 2.7. Determination of the Probiotic Bacteria Amount in Fish Intestinal Contents

The intestines were cooled and transported to the laboratory within 2 h. The posterior intestine was separated using sterile instruments, and intestinal contents were extracted into a microtube. Contents from the intestines of five individuals were pooled, homogenized, and averaged.

Microbiological analysis was performed to estimate both vegetative forms of bacilli and spores. To count total bacilli, undiluted samples were serially diluted (1:10 in sterile saline), plated on LB agar, and incubated 48 h at 37 °C. For spore count, homogenates were pasteurized (95 °C, 5 min) to kill vegetative cells, followed by dilution and plating. Colonies were counted after 48 h. Spore counts were derived from post-pasteurization plates, while total bacilli (vegetative and spores) were enumerated from pre-pasteurization plates. Colony morphology (wrinkled, dry, opaque) and Gram staining (rod-shaped, spore-forming) were used to confirm Bacillus identity. Vegetative cell counts were calculated as total CFU—spore CFU.

### 2.8. Genomic DNA Extraction, Sequencing, Assembly and Annotation

DNA was isolated according to the protocol described by Gautam [63]. Sequencing was performed on the MinION device using the Rapid Barcoding Sequencing Kit V14 reagent kit in accordance with the instructions. Quality control of the readings was carried out using the NanoPlot v1.42.0 program, and filtering and rejection of low-quality readings using the Filtlong v0.2.1 (—min_length 200; —keep_percent 90), discarding the worst 10% of bases by quality and retaining reads ≥ 200 bp.

Genomes were assembled using Flye 2.9.5 with “—nano-hq” flag. The quality of the genome assembly was assessed using the CheckM v1.2.3 software (assessment based on the presence/absence of single-copy genes) and CheckM2 v1.0.2 (assessment program based on machine learning) and by visual assessment of the assemblage to the ring chromosome using Bandage v0.9.0. The circular genome map was built using the Proksee service [64]. The annotation was conducted using Bakta v1.8.2 (DB: v5.0—Light) [65].

The analysis of antibiotic resistance genes was carried out using CARD RGI (The Comprehensive Antibiotic Resistance Database (CARD) Resistance Gene Identifier (RGI)) [66]. The functional analysis of the metabolic pathways was performed using Blast KOALA [67].

The analysis of genes for the synthesis of secondary metabolites was carried out using AntiSMASH with “—relaxed” detection strictness parameter [68].

### 2.9. Phylogenetic Analysis

The taxonomic identification was performed on the basis of ANI (Average Nucleotide Identity), according to the GTDB taxonomy (Genome Taxonomy Database) using the classify_wf GTDB-Tk protocol v4.2.0 [69] based on The Genome Taxonomy Database (GTDB) (release 220) [70]. The phylogenetic tree was built using the de_novo_wf protocol based on concatenated alignment with 120 phylogenetically informative markers. The tree was visualized using the Interactive Tree of Life (iTOL) online [71].

### 2.10. Analysis of the Differential Expression in Carp Tissues

Fragments of carp muscle tissue were used to analyze the differential expression of genes. The analysis was performed in four biological replicates (cDNA from the muscles of different fish) and three technical (cDNA from the muscles of one fish). Fish were divided by age into fry (14 days from the start of active nutrition) and adults (one month from the start of active nutrition). Based on probiotic treatment, two groups were formed: Group 1 (strains MT14 + MT42) and Group 2 (strains MT141 + MT142). During transportation and storage, tissue fragments were fixed using the IntactRNA reagent (Evrogen, Moscow, Russia), and subsequent RNA isolation was performed using the ExtractRNA kit (Evrogen, Moscow, Russia) in accordance with the manufacturer’s recommendations. The reverse transcription was performed using the MMLV RT kit (Evrogen, Moscow, Russia) in accordance with the manufacturer’s recommendation. The resulting cDNA was analyzed by real-time PCR using a 5X qPCRmix-HS kit (Evrogen, Moscow, Russia) on the RotorGene Q device (Qiagen, Hilden, Germany). Several stress- and immunity-related genes were investigated, namely *cxc* (CXC chemokine gene), *lyz* (lysozyme gene), *hsp70* (heat shock protein 70 gene), *il-1β* (interleukin 1 beta gene), *il-10* (interleukin 10 gene), *igf-1* (insulin-like growth factor 1 gene), *gst* (glutathione S-transferase gene), *β2m* (beta 2 microglobulin gene), and *mt1* (metallothionein 1 gene); *act* (actin 1 beta gene) was used as the reference gene. The primers used in the study are shown in Table 1.

Reaction conditions were optimized in accordance with the selected primers and kit guidelines. Differential expression of target genes was evaluated using 2^−ΔΔCt^) method.

### 2.11. Statistical Analysis

Data normality was verified using the Shapiro–Wilk test, statistical significance was determined by two-tailed Student’s *t*-test (for normally distributed data) and the Wilcoxon test (if the data distribution was non- normal). No multiple testing corrections were applied in gene-expression analyses. Visualization of the expression data was performed using the ggplot2 package. All calculations were performed in Rstudio (R 4.2.1).

## 3. Results

### 3.1. In Vitro Screening and Selection of Probiotic Strains

To identify strains that possess at least one of the potential probiotic properties of interest, suspension from bottom sediments was inoculated immediately onto dishes with test media to determine enzymatic activity. A total of 51 strains, secreting exoproteases, and 52 strains, secreting exoamylases, were isolated. They were then cross-tested for both types of activity.

Of the 103 strains selected at the first stage, only 14 strains exhibited high exolytic activity: four strains for one type activity, six strains for one type activity and four strains for all three type activities (Table 2). Some of the strains had only high proteolytic or only amylolytic activity, some combined both types of activity. For data conciseness, Table 2 presents only strains with a clearance zone around the colony greater than 8 mm on at least one test medium, alongside reference probiotic strains *B. amyloliquefaciens* B1895 and *B. subtilis* KATMIRA. They were included since in our prior studies they demonstrated probiotic effects in poultry [51,52] and fish [53].

All strains listed in Table 2 were tested for antibiotic resistance (against azithromycin; amoxicillin/clavulanate; ampicillin; tetracycline; gentamicin; oxacillin; clarithromycin; cefazolin; ceftriaxone) and hemolysis. All strains were found to be safe for further use in aquaculture.

**Figure 1:**
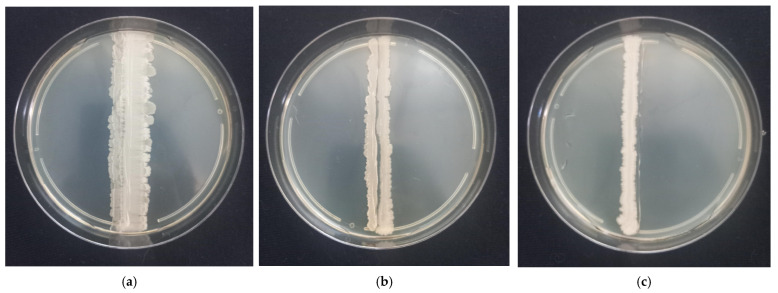
Interaction of *Bacillus* strains: (**a**). synergism (right: MT42, left: MT14); (**b**). neutrality (right: MT42, left: MT27), (**c**). antagonism (right: MT102, left: MT27).

To assess the possibility of their joint use in one multi-strain preparation, pairwise co-culture assays were conducted on nutrient agar plates. Strains were inoculated 5 mm apart from each other to allow the colonies to fully develop. Plates were incubated at optimal growth conditions for 48 h without pre-drying to facilitate unrestricted colony development. The resulting interactions could be divided into three types (Table 3):

1. Synergistic Merging: Colonies exhibited unrestricted growth into each other’s territories, forming confluent biomass without visible separation zone (Figure 1a, marked with “+” in Table 3);
2. Neutral Coexistence: Colonies halted growth approximately 0.5–1 mm apart, while continuing to grow in opposite direction (Figure 1b, marked with “=” in Table 3)
3. Antagonism: Growth suppression at the interaction interface, characterized by reduced biomass density or partial clearance zones (Figure 1c, marked with “−” in Table 3).

Of 182 pairwise combinations, 22 showed synergy, 112 neutrality, and 48 antagonism.

When developing the multistrain formulation, we avoided antagonistic pairs and prioritized synergistic pairs over neutrally interacting ones.

Among the strains with enzymatic activity, strains with both high exoamylase and exoprotease activity were selected. Among these six strains (MT14, MT27, MT42 and MT74, MT141, MT142), pairs MT14 + MT42 and MT141 + MT142 were selected, since they were able to form a joint biofilm.

### 3.2. Analysis of the Results of the Whole-Genome Sequencing

As a result of the ONT sequencing and bioinformatic reading processing, full-genomic nucleotide sequences of 4 strains—MT14, MT42, MT141, and MT42—were obtained, with 26× coverage for MT14, 81× coverage for MT42, 54× coverage for MT141, 83× coverage for MT142. Assembly statistics obtained in the CheckM2 and Bakta programs is presented in Table 4.

**Figure 2:**
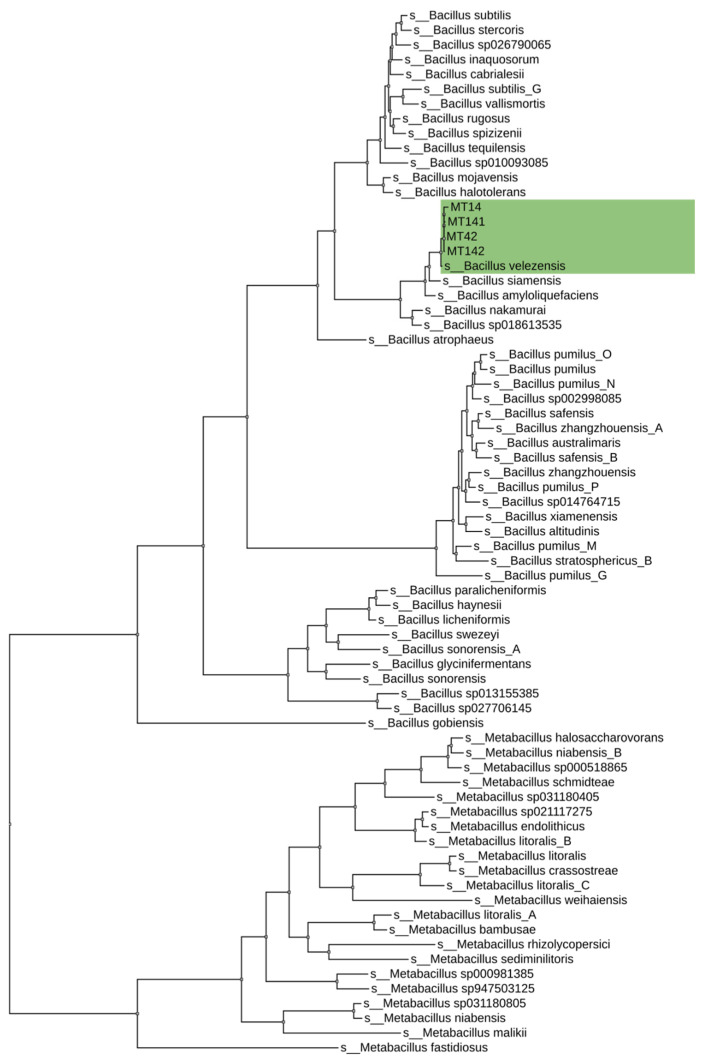
The phylogenetic tree for the studied strains and reference genomes from the GTDB database, based on the alignment of 120 taxonomically significant markers. The genus *Metabacillus* was selected as an outgroup for comparison. The strains used in this study, together with reference *B. velezensis* strain, are highlighted in green.

The results of taxonomic identification using GTDB-tk based on ANI assigned all four strains to *Bacillus velezensis*. The taxonomic affiliation of the strains is shown in Figure 2.

**Figure 3:**
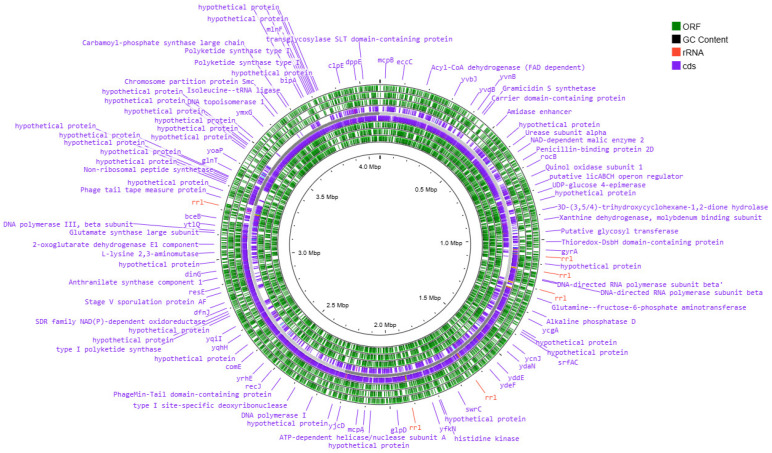
Circular graphical map of *Bacillus velezensis* MT14. From outer to inner rings: ORFs on the forward strand, CDS on the forward strand, CDS on the reverse strand, ORFs on the reverse strand, GC content, contig(s). Ribosomal RNA genes are marked in red between all other CDS (violet), and ORFs are green.

**Figure 4:**
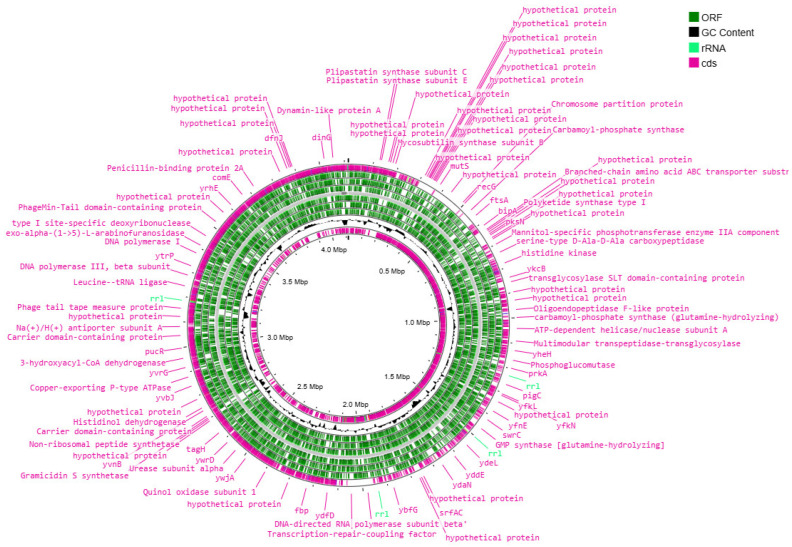
Circular graphical map of *Bacillus velezensis* MT42. From outer to inner rings: ORFs on the forward strand, CDS on the forward strand, CDS on the reverse strand, ORFs on the reverse strand, GC content, contig(s). Ribosomal RNA genes are marked in cyan between all other CDS (purple), and ORFs are green.

**Figure 5:**
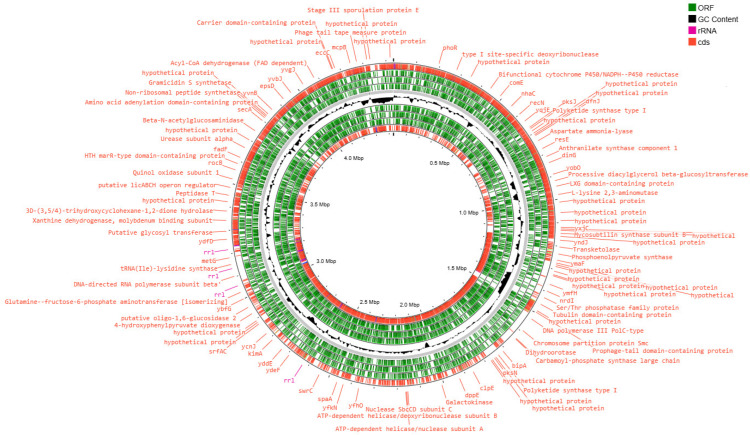
Circular graphical map of *Bacillus velezensis* MT141. From outer to inner rings: ORFs on the forward strand, CDS on the forward strand, CDS on the reverse strand, ORFs on the reverse strand, GC content, contig(s). Ribosomal RNA genes are marked in purple between all other CDS (orange), and ORFs are green.

**Figure 6:**
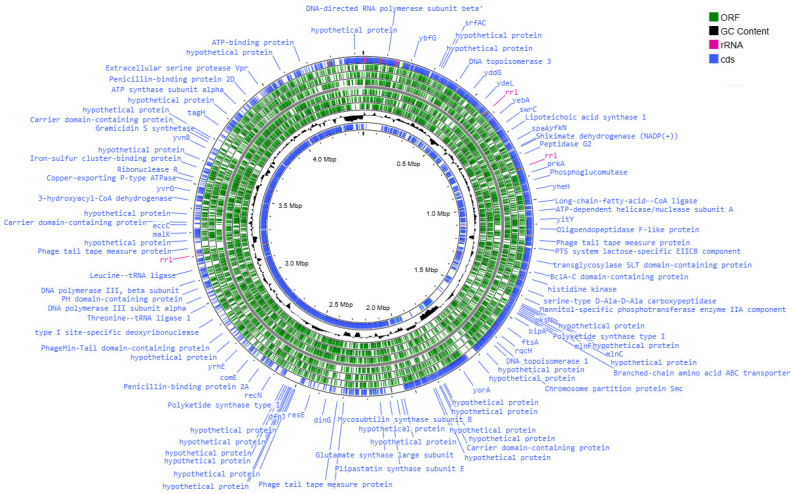
Circular graphical map of *Bacillus velezensis* MT142. From outer to inner rings: ORFs on the forward strand, CDS on the forward strand, CDS on the reverse strand, ORFs on the reverse strand, GC content, contig(s). Ribosomal RNA genes are marked in purple between all other CDS (blue), and ORFs are green.

The genetic apparatus of these bacteria is represented by the ring chromosome, the visualization of which is shown in Figure 3, Figure 4, Figure 5 and Figure 6.

The genome assembly quality was assessed using CheckM, which revealed highly complete and minimally contaminated drafts for all four strains. MT42, MT141, and MT142 exhibited near-complete assemblies with >99.9% completeness and low contamination (1.19–2.32%), while MT14 showed slightly reduced completeness (97.35%) but remained within acceptable limits for high-quality drafts. All assemblies displayed strong structural integrity, with full rRNA operons, complete tRNA sets further supporting their reliability for downstream genomic analyses. The high contiguity (N50 > 4 Mbp for MT42, MT141, and MT142 and N50 > 1Mb) and low contamination confirm that these assemblies are well-suited for comparative genomics and functional studies.

### 3.3. Functional Analysis of Genomes

**Figure 7:**
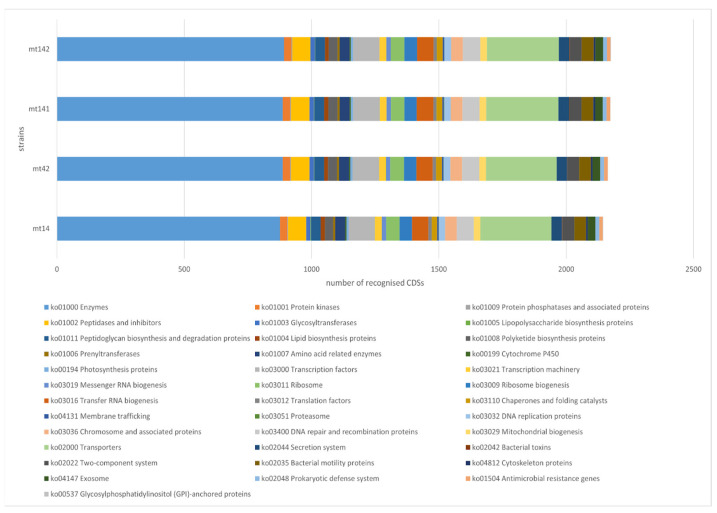
Groups of genes of strains MT14–142 annotated using KEGG Mapper Reconstruct.

The functional annotation of strain genomes was performed using KEGG Annotation—BlastKOALA. Of the 4571 CDSs of the MT 14 strain, 2266 were annotated (49.5%); of the 4433 CDSs of the MT 42 strain, 2292 were annotated (51.7%); of the 4918 CDSs of the MT 141 strain, 2331 were annotated (47.3%); of the 4813 CDSs of the MT 142 strain, 2332 were annotated (48.4%) with KO identifiers of the KEGG database. Based on the obtained data, the functional groups of genes of the studied strains were reconstructed using KEGG Mapper Reconstruct (Figure 7).

The dominant functional categories were Enzymes (ko01000) and Transporters (ko02000), which had the highest numbers across all samples (879–896 and 279–285 CDSs, respectively). Other well-represented pathways included Transcription factors (ko03000), DNA repair and recombination proteins (ko03400), and Ribosome-related functions (ko03011/ko03009). There are no significant differences in KEGG functional categories between the strains. The vast majority of categories show identical or nearly identical CDS counts; variations are minimal and unlikely to be biologically meaningful.

#### 3.3.1. Genes of Probiotic Properties

The genes that can determine probiotic traits were analyzed relying primarily on three functional categories: (1) providing resistance under stressful conditions.; (2) having antimicrobial properties; (3) improving adhesion and/or biofilm formation.

**Figure 8:**
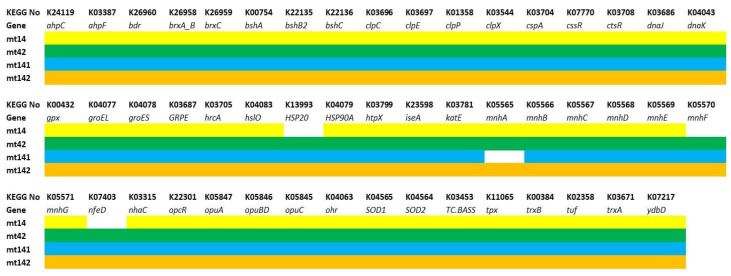
Stress response genes annotated in MT14–142 strains using KEGG Annotation—BlastKOALA.

The first group included genes providing antioxidant effects (peroxidases—*ahpC-F*, *gpx*, *ohr*, *tpx*; catalases—*katE*, *ydbD*; superoxide dismutases—*SOD1-2*; thioredoxins—*trxA-B*; bacilliredoxins specific for Gram-positive bacteria—*brxA_B-C*; and bacillithiols—*bdr*, *bshA-C*), maintain proteome stability (temperature shock proteins and chaperones—*cspA, hslO*, *HSP20*, *HSP90A*, *htpX*, *dnaJ*, *dnaK*, *groEL*, *groES*, *GRPE*; intracellular proteases—*clpC-X*), provide adaptation to gastrointestinal conditions (membrane pumps-regulators of pH homeostasis—*mnhA-E*, *nhaCl*; bile acid transporters—*TC.BASS*; osmoprotectors—*opuA-D*), as well as the corresponding regulatory genes (*cssR*, *ctsR*, *hrcA*, *opcR*, *tuf*) (Figure 8).

The MT14 strain lacks nfeD, which is responsible for membrane-associated serine proteases, the heat shock protein HSP20, and mnhF (multicomponent Na+:H+ antiporter subunit F). The MT42 strain lacks mnhA (multicomponent Na+:H+ antiporter subunit A). These deficiencies may slightly reduce the resistance of strains MT14 and MT42 to acid and alkaline stress.

The analysis shows that all four strains have key genes for resistance to thermal, osmotic, and oxidative stress, as well as to acid and bile, which is important for survival in the gastrointestinal tract.

**Figure 9:**
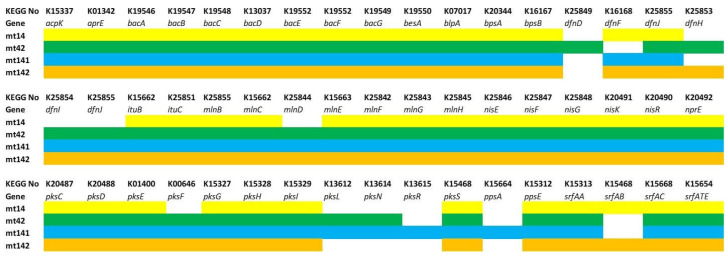
Antimicrobial genes annotated in MT14–142 strains using KEGG Annotation—BlastKOALA.

Genes responsible for the synthesis of antimicrobial metabolites (polyketides—*acpK*, *bacA-G*, *dfnD-J*, *mlnB-H*, *pksC-S*; non-ribosomal peptides and lipopeptides—*besA*, *bpsA-B, ituB-C*, *nisE-R*, *ppsA-E*, *srfAA-ATE*) and bacteriocins (subtilisin—*aprE*; bacillolysin—*NprE*) are presented in Figure 9.

All strains have been found to have genetic markers suggesting their ability to synthesize bacillolysin, subtilisin. The synthesis of plipastatin in MT14, MT42, and MT142 is disrupted due to the absence of the key gene ppsA. All strains lack at least one dnf gene (difficidin), and all except MT141 lack at least one pks gene (bacillaene). However, this could be a false-negative result associated with the high variability of PKS genes, which include the aforementioned genes.

**Figure 10:**
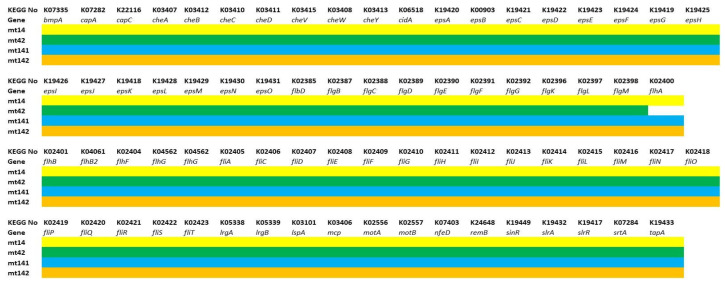
Adhesion, motility, and biofilm formation genes annotated in MT14–142 strains using KEGG Annotation—BlastKOALA.

The third group included genes responsible for adhesion (*bmpA*, *nfeD*, *srtA*, *tapA*), extracellular matrix formation (polyglutamic acid synthesis—*capA-C*; exopolysaccharide—*epsA-O*), ensuring cell mobility (flagella assembly and function—*fdbD*, *flgB-M*, *flhA-G*, *fliA-T*, *motA-B*; chemotaxis—*cheA-Y*; *mcp*) and biofilm population density control (*cidA, lrgA-B*), and regulatory genes (*remB*, *sinR*, *sirA-R*) (Figure 10).

The strains revealed an almost identical set of genes responsible for the formation and functioning of flagella, chemotaxis, extracellular matrix synthesis, and adhesion. The MT42 strain lacks the flagellar biosynthesis protein FlhA. However, it is difficult to say how much this will affect the motility of the strain.The presence of these genes suggests that the strains have the ability to effectively form biofilms and colonize the gastrointestinal tract of the host organism.

Thereby, the strains have genes for resistance to stress and adaptation to the conditions of the gastrointestinal tract. Their genomes contain adhesion and chemotaxis genes, which are important for colonization of the intestine. The presence of genes for the biosynthesis of secondary metabolites indicates the ability to synthesize antimicrobial compounds and, thus, show antagonism against pathogenic microorganisms.

#### 3.3.2. Genes of Lytic Enzymes

The genes of hydrolytic enzymes (cellulases, proteases, chitinases) were also analyzed (Table 5).

The highest number of endoglucanase copies (two) was observed in strain MT141, while the other strains had one copy each. Among proteases, the highest number of copies was found in strain MT42, with four copies of various hemolysins. Strain MT142 possessed two copies of a CwlT-like protein with a lysozyme domain, while the other strains had one copy. Other enzymes with a high copy number included 6-phospho-beta-glucosidase (four copies in MT14 and MT142) and maltose-6′-phosphate glucosidase (three copies in MT141). The data demonstrate differences in the genetic potential of the strains related to the synthesis of lytic enzymes.

All strains had endoglucanase genes in their genomes, and some had other cellulase genes (MT42, MT142). These enzymes are crucial for probiotics that facilitate the digestibility of plant-based feeds in aquaculture [72,73].

Core metabolic enzymes identified in all or almost all strains, such as alpha-amylases, beta-glucanases and glucosidases, glucan hydrolases, which are involved in the assimilation of complex polysaccharides.

Cellulases, found only in MT42 and MT142, together with endoglucanases provide these probiotics ability to degrade cellulose. Cellulases break down cellulose while endoglucanases break the internal β-glycoside bonds in cellulose to facilitate further cleavage by other enzymes.

Lytic enzymes include lysozyme family proteins and CwlT-like proteins with a lysozyme domain. Their antimicrobial activity is due to the hydrolysis of peptidoglycans. CwlT-like proteins cleave the bonds between N-acetylmuramic acid (NAM) and N-acetylglucosamine (NAG) in the peptidoglycan of gram-positive bacteria.

Levansaccharase, which is present in all the studied strains, produces fructo-oligosaccharides, which are prebiotics for lactobacilli [74]. Thus, the enzymatic profile of these four strains provides effective breakdown of complex carbohydrates, cellulose, and cellular structures to improve digestion, protect the body from pathogens, and adapt to aquaculture conditions.

### 3.4. Antibiotic Resistance Gene Screening

The genomes of the strains were additionally analyzed for the presence of antibiotic resistance genes to assess their biosafety for potential use. Although several clusters of resistance genes have been found, as mentioned above, the data from in vivo tests demonstrate the sensitivity of these bacteria to the main classes of antibiotics. This suggests that the detected resistance genes may be non-functional or epigenetically repressed.

Further analysis using KEGG mapper revealed that most of these clusters of resistance genes are incomplete (Table 6).

The analysis of four MT strains revealed several clusters of antimicrobial resistance (AMR) genes, including both complete and incomplete orthologous groups from the KEGG database. *AbcA* (M00700), encoding an ABC-type efflux pump, was fully conserved (2/2 blocks) in all strains. Among other determinants of multidrug resistance, efflux pumps *norB* (M00702) and *qacA* (M00714) were identified, each missing half of their functional domains (1 of 2 blocks). Partial deletions (missing 2 of 3 blocks) were observed in all strains in the *bla* (M00627) system, responsible for beta-lactam antibiotic resistance. The *tet38* (M00704) gene, encoding a tetracycline efflux pump, contained only one of the two required blocks. The *mprF* (M00726) cluster, responsible for resistance to cationic antibiotics, was also incomplete (2 of 3 blocks were missing).

These findings imply that although the presence of partial resistance clusters indicates the need for strain control in biotechnological production to prevent the acquisition of missing genes through horizontal transfer, in general, fully functional clusters of resistance genes are limited to non-specific efflux pumps, which are characteristic of many bacteria and do not pose significant safety risks [75].

#### Secondary Metabolite Production: Non-Ribosomal Peptides and Polyketides

To identify genetic determinants of probiotic activity, we analyzed the genomes for biosynthetic gene clusters (BGCs) associated with bioactive compounds. This included genes encoding non-ribosomal peptides (NRPs) with antimicrobial, antifungal, or immunomodulatory properties, as well as polyketides and other secondary metabolites.

The identified secondary metabolites are summarized in Table 7. The numbers indicate the percentage similarity of the detected substance to reference in AntiSMASH database.

These metabolites can provide some of the effects of probiotics in aquaculture: to suppress harmful bacteria and fungi, enhance the immune responses of fish, increasing resistance to infections.

It can be noted that 100% of the analyzed strains carry the genes for the synthesis of bacilysin and bacillaene. However, the presence of these genes does not yet ensure their synthesis, since, as shown by us [83] and other authors [84,85], the expression of NPP genes may strongly depend on environmental conditions.

While most strains share 100% similarity for bacillaene (except MT142 at 71%), MT14 stands out with an unusually low 8% similarity for difficidin, contrasting sharply with the >90% seen in other strains. MT42 uniquely carries a 100% identical macrolactin cluster, whereas the remaining strains range about 90%. Additionally, mycosubtilin is fully conserved in MT14 and MT141 but absent in MT42 and MT142, while bacillomycin shows the opposite pattern—missing in MT14 and MT141 but present at 100% in MT42 and MT142. These disparities suggest strain-specific adaptations that could influence their bioactive potential, particularly in antimicrobial, antiviral, and antifungal functions.

In general, the production of these bioactive compounds makes these strains of *B. velezensis* and *B. subtilis* promising probiotics for improving the health and productivity of aquaculture facilities. However, there is a possibility that their antimicrobial or immunostimulating effect is not detectable in vitro but is present in vivo. The study of this will be the subject of our further research.

### 3.5. Fish Growth Parameters

Two probiotic formulations were developed based on 1) *B. velezensis* MT14 and MT42 strains, and 2) *B. velezensis* MT141 and MT142, which were added to the feed during the manufacturing process.

The final probiotic cells count in the feed was 3.7 ± 0.4·10^6^ CFU/g for MT42 + MT14, and 6.4 ± 0.3·10^6^ CFU/g for MT141 + MT142.

During in vivo studies, assessment of growth, size, feed efficiency, and survival in carp subjected to different probiotic treatments was conducted, allowing for a detailed comparison of the effects of each probiotic formulation on fish performance.

As it can be seen from the results provided in Table 8 and Table 9, supplementation of carp diets with probiotic preparations led to notable improvements in several key fish-breeding performance indicators compared to the control group at the end of the experiment.

During the experiment, optimal water quality parameters were observed. The concentration of nitrite nitrogen was below the detection limit (<0.005 mg/L). The concentration of ammonium nitrogen did not exceed 0.04 mg/L in all treatments. The concentration of nitrate nitrogen did not exceed 0.013 mg/L in all tanks. The level of dissolved oxygen in water did not decrease below 8 mg/L due to constant aeration and water quality control.

The average individual weight at the end of the experiment was significantly higher in both experimental groups compared to the control (*p* < 0.05). Group 2 (MT141 + MT142) exhibited a 56.62% higher biomass increase than in the control group, while Group 1 (MT14 + MT42) exhibited a 40.75% higher biomass growth (Table 8 and Table 9). However, no statistically significant difference was observed between experimental groups 1 (MT14 + MT42) and 2 (MT141 + MT142).

**Figure 11:**
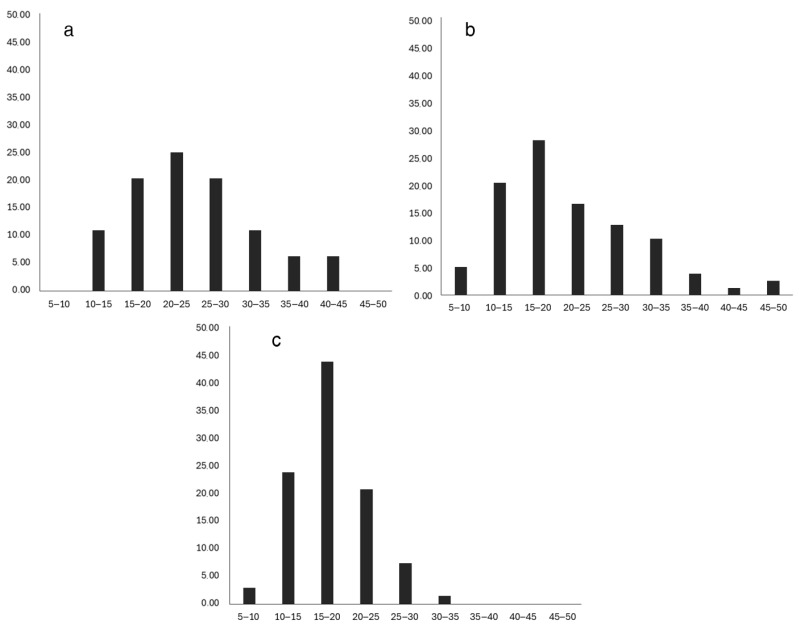
Distribution of juvenile carp *Cyprinus carpio* Linnaeus, 1758 into nine size groups: axis X–size group (g), axis Y–share of individuals in the size group (%): (**a**)—experimental group No. 1 (MT14 + MT42), (**b**)—experimental group No. 2 (MT141 + MT142), (**c**)—control group.

Differences in size distribution were observed (Figure 11). In experimental Group 1 (Figure 11a), individuals with a body weight of 20 to 25 g represented the largest proportion (25.00%). Individuals with a body weight in the range of 15–20 g and 25–30 g (20.31% each) were less common. At the end of the experiment, no individuals with a body size ≤ 10 g were recorded in the first group. In experimental Group 2 (Figure 11b), 5.06% of the individuals had a body weight of less than 10 g. The size of 15–20 g was more common than others, accounting for 27.85% of the population. In the control group (Figure 11c), the proportion of individuals with a body weight ≤ 10 g was 2.96%, and the size group of 15–20 g was 43.7%.

According to a number of other performance metrics (individual biomass increase, %; specific growth rate, feed conversion rate), these strains proved to be promising for use in aquaculture. Both probiotic groups demonstrated enhanced growth performance, feed efficiency, and survival compared to the control, with Group 2 showing the highest values in several parameters.

### 3.6. Microbiological Tracking of Probiotic Colonization

To track probiotic colonization, we compared colonies from intestinal content cultures to reference plates of pure probiotic strains. Probiotic-derived colonies were identified by their morphological congruence with reference cultures and their absence in control groups receiving alternative probiotics. Their characteristic morphological features were the shape of the surface and edges, the location of wrinkles, and the production of pigment in the medium.

Quantitative analysis revealed significant colonization by the administered probiotic strains in treated carp (Table 10). Minor amounts of other bacilli with a different morphology were also found in the intestinal contents, but their number did not exceed 10^4^ CFU/g.

A sufficiently high number of bacilli of various morphologies was observed in the control (a total of eight different morphotypes). They are likely representing native microbiota of catfish acquired through environmental exposure (water and fee). In contrast, probiotic-supplemented carp exhibited a near-exclusive dominance of two morphotypes, consistent with the administered *B. velezensis* strains (MT14 + MT42 or MT141 + MT142). Minor inclusions of other morphotypes were rare, confirming effective displacement of native bacilli by the probiotics.

Despite no net increase in total bacilli compared to feed concentrations, spore-to-vegetative cell conversion rates reached 94% (Group 1: MT14 + MT42) and 95% (Group 2: MT141 + MT142). This indicates that most spores germinated into metabolically active cells within the carp intestine, enabling sustained probiotic function.

### 3.7. Analysis of the Differential Expression of Fish Genes

**Figure 12:**
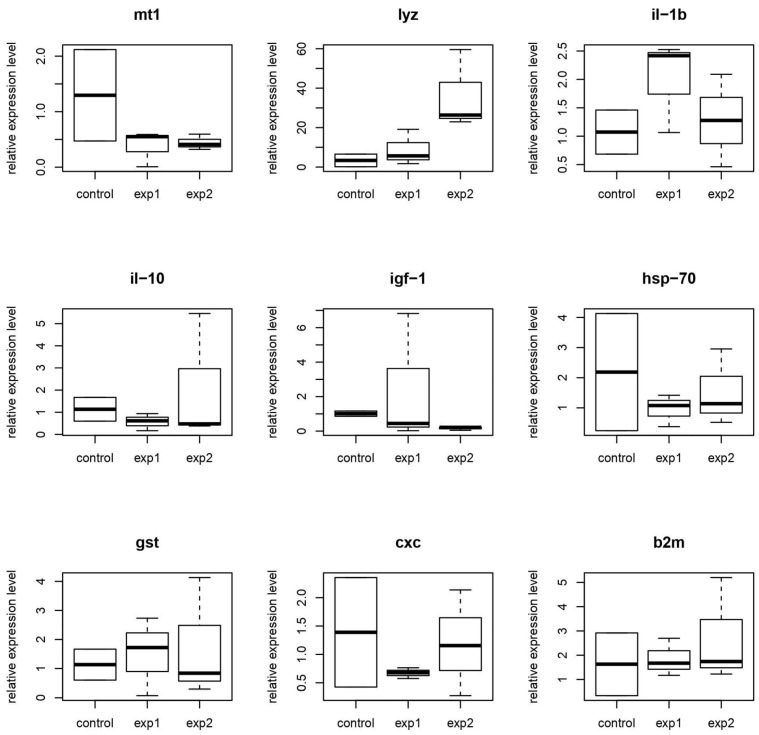
Relative gene expression rates in the muscle tissue of the full-grown *C. carpio*. No statistically significant changes were observed in group 1 relative to the control group (*p* > 0.05).

**Figure 13:**
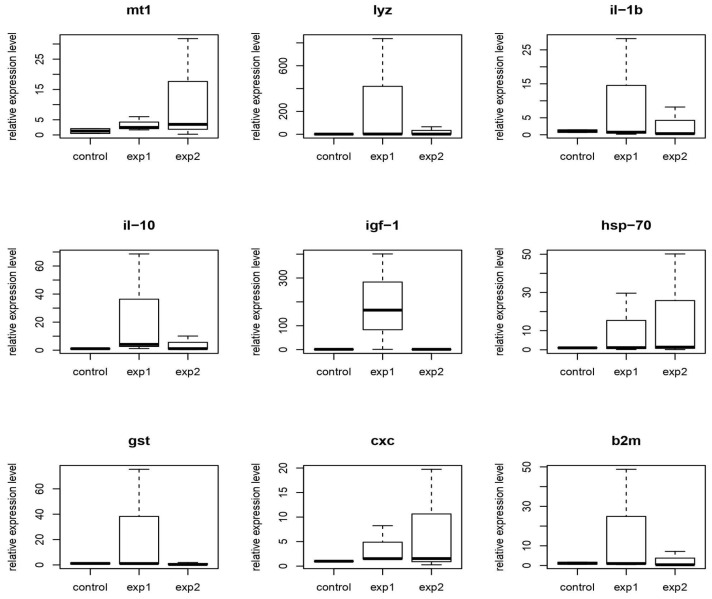
Relative gene expression rates in the muscle tissue of the mature *C. carpio*. No statistically significant changes in expression level were observed for the studied genes relative to the control group (*p* > 0.05).

Results of the differential expression analysis of genes is shown in Figure 12 (carp model) and Figure 13 (full-grown fish).

There are no statistically significant results (*p* > 0.05) of changes in the expression of most genes in the muscles of carp fry in group 1, while group 2 demonstrated an increase in *lyz* gene expression and a decrease in *igf-1* expression (*p* < 0.05).

There are no statistically significant changes in expression in the muscles of full-grown carp.

The data suggest that probiotics have a greater effect on carp fry than on adults. A probiotic based on MT141 and MT142 strains had a minor effect on gene expression only in carp fry. This contrasts with our earlier findings in African catfish *Clarias gariepinus*, where it was shown that probiotics can cause significant changes in the expression of some of the listed genes [86]. The disparity implies that the mechanisms underlying these probiotics’ action are different, potentially involving enzymatic activity, nutrient absorption, or competitive exclusion of pathogens rather than direct transcriptional modulation.

## 4. Discussion

Using the pipeline described above, we have succeeded to identify and characterize novel *Bacillus velezensis* strains with enzymatic activity to improve the digestibility of feeds in carp aquaculture. The most notable result was an increase in average individual weight in carp receiving the MT14 + MT42 probiotic supplement compared to controls, demonstrating the potential of these strains to enhance growth and feed efficiency.

### 4.1. Bacilli as Probiotics for Aquaculture

Probiotics play an important role in animal husbandry and poultry farming, where their use has long been shown to increase productivity and reduce the incidence of livestock [87,88,89]. However, probiotics in aquaculture are less common and often have less effect than in warm-blooded animals [12]. This can largely be attributed to the fact that many probiotics used in aquaculture are derived from animal husbandry, without sufficient consideration of the physiological and ecological differences between aquatic and terrestrial hosts. But fish differ significantly from warm-blooded animals in terms of physiological parameters and feeding preferences, as well as vulnerabilities and requirements [90,91,92]

Carp specifically has its own maintenance features. The carp (*Cyprinus carpio*), like other members of the Cyprinidae family, is a stomachless fish (agromastric species). Its digestive system includes an expanded cardiac section of the intestine (morphologically similar to the stomach, but not secreting pepsin and hydrochloric acid), which is typical of phylogenetically ancient fish [93]. Notably, a long intestine compensates for limited stomach digestion, aiding nutrient uptake [94].

Recognizing these differences, we selected bottom sediments as the source of potential probiotic strains. We consider the “fish–water–bottom sediments” system, wherein microorganisms excreted from the fish’s body (primarily through feces) are incorporated into the bottom sediments. Subsequently, these microbes can re-enter fish intestines via water movement, gill ventilation, ingestion of water, and through their association with food particles, thereby facilitating their reintroduction into the fish gut. So, the microbiota of bottom sediments and the intestines of aquatic organisms largely overlap. Studies show that 27.8% of shrimp intestinal microbiota overlaps with the microbial composition of bottom sediments. In fish, this percentage can reach up to 71.7%. It has been demonstrated that sediment microbiota is the primary source of fish microbiota, directly shaping the microbiome, especially for benthic fish. Strains derived from bottom sediments successfully colonize the host’s intestine [95,96]. Therefore, we hypothesize that bacteria isolated from bottom sediments will be better adapted to life in the intestines of fish than bacteria isolated from other sources.

Genomic analysis revealed that all four strains belong to the species *Bacillus velezensis*. This species is characterized by a wide range of antimicrobial activity against various pathogens [97,98], the ability to produce many lytic enzymes such as protease, cellulase, amylase, and glucanase [48], the ability to improve blood parameters related to inflammation and immunity as well as improve the stability of the intestinal microbiota and the integrity of the gut barrier [98].

The mechanisms underlying *Bacillus* probiotics benefits are multifaceted. Firstly, this probiotic has been shown to enhance the activity of digestive enzymes in aquatic animals. Secondly, it demonstrated stimulation of immunity and antagonism to pathogens. Finally, the production of its own enzymes allows it to improve the digestion of dietary components by the host.

Our findings suggest that our strains exhibit at least some of those effects. Digestive enzyme enhancement was clearly demonstrated in our in vitro screening, where strains MT14, MT42, MT141, and MT142 produced substantial clearance zones on test media—proteolytic activity with clearance zones of 5–11 mm and cellulolytic activity with clearance zones of 14–17 mm—suggesting these strains can effectively break down complex plant polysaccharides in fish diets.

Endogenous enzyme production capacity is also supported by our genomic analysis, which identified numerous lytic enzyme genes in all four strains. The genomes contained multiple copies of glycosyl hydrolases, proteases, and cellulases. The presence of these genes explains the observed growth enhancement (31.9–42.1% increase in individual biomass) despite modest improvements in feed conversion ratio, which will be discussed in more detail below.

Immune stimulation mechanisms, while not directly evidenced in our muscle tissue gene expression analysis, may still be occurring in other immunologically relevant tissues. Although we observed no statistically significant changes in expression of immune-related genes (*cxc*, *il-1β*, *il-10*, *hsp70*) in muscle samples, it’s important to note that immune modulation by *Bacillus* probiotics can also manifest in tissues with higher immunological activity such as spleen, head kidney, or intestinal mucosa [99].

Although we used muscle tissue for gene expression analysis, which aligns with established practices in fish nutritional research for assessing growth-related effects [100,101], future studies should examine gene expression in these tissues. The slight increase in survival rate in our experimental groups (97.33% vs. 94.67% in control) may reflect subtle immune benefits not captured in our muscle tissue analysis.

### 4.2. Enzymatic Activity and Its Role in Improving Feed Digestibility

The introduction of probiotics with the potential to produce various hydrolytic enzymes, including cellulase, protease, and amylase, can improve the digestibility of feed components and enhance nutrient absorption [30,36,37,38,40]. Bacteria of the genus *Bacillus* are able to secrete various hydrolytic enzymes, such as β-1,3-glucanases, proteases, and cellulases, which helps to improve the digestive processes in fish [19,102]. Various studies confirm the ability of various *Bacillus* strains to produce key enzymes for the proper digestion of feed [35,103] and high proteolytic activity of *B. amyloliquefaciens* and *B. subtilis*, which secrete proteases, amylases, and lipases [103]. The work [35] expands this list to include such enzymes as α-glucosidase, naphthol-AS-BI-phosphohydrolase, esterase lipase, acid and alkaline phosphatases, caseinase and lecithinase [35]. A study [104] demonstrated the positive effect of *B. safensis* NPUST1 on the Nile tilapia *Oreochromis niloticus* (Linnaeus, 1758), increasing feed efficiency and improving the growth rate of individuals [104]. Another study experimentally confirmed the ability of *B. cereus* YB1 to increase the activity of protease, amylase, and lipase in juvenile black sea bass *Sebastes schlegelii* Hilgendorf, 1880, which led to a noticeable increase in growth rates [98].

Our goal was to obtain a multi-strain antimicrobial preparation with lytic properties. We focused on the production of exogenous proteolytic, amylolytic, and cellulolytic enzymes [105]. Media used in screening (containing milk, starch, and carboxymethyl cellulose) clearly show the lytic activity via a light zone around the colony.

Among all isolated strains, 14 had a high degree of exolytic activity (Table 1), of which nine had all studied activities simultaneously, and therefore were selected for enhancing feed digestion in fish.

We used multi-strain probiotic drugs to broaden enzymatic diversity and functional synergy. However, bacilli can inhibit each other through the production of antimicrobial compounds and various toxin-antitoxin systems [106]. Therefore, it was necessary to perform compatibility testing. Strain-specific interactions resulted in some pairs showing mutual growth inhibition, while others formed stable biofilms when co-cultured (Table 2). Two compatible pairs of strains were determined and used in Groups 1 and 2, respectively.

Functional enzyme genes (endoglucanases, cellulases, alpha-amylases, beta-gluconases and glucosidases, glucan hydrolases, lysozyme family proteins and CwlT-like proteins with a lysozyme domain) were found in the genome of our strains, which coincides with in vitro test data. This confirms the effectiveness of chosen screening methodology.

Proteolytic activity—the clearance zones observed on milk medium (8–11 mm) directly correspond to the presence of multiple protease genes identified in the genome sequences of all four strains.

Amylolytic activity—the starch degradation zones (5–11 mm) correlate with the presence of alpha-amylase family genes, alpha-amylases, and glucosidase/amylase genes in the genomes.

Cellulolytic activity—the substantial clearance zones on carboxymethylcellulose medium (14–17 mm) align with the presence of endoglucanase genes in all strains and cellulase genes in MT42 and MT142.

The genomic analysis revealed strain-specific patterns in enzyme gene distribution that perfectly matched the observed phenotypic differences in enzymatic activity. Strains with higher proteolytic activity in vitro (MT141, MT142) showed more copies of glycosyl hydrolases of the alpha-amylase family. Strains with stronger amylolytic activity (MT42) contained more copies of beta-glucanase and 6-beta-D-glucan-glucanohydrolase genes.

The exceptional cellulolytic activity observed in MT42 and MT142 was confirmed by the presence of dedicated cellulase genes in these strains only, while all strains possessed endoglucanase genes.

Levansaccharase, which is also present in all the studied strains, produces fructo-oligosaccharides, which are prebiotics for lactobacilli [74]. Studies show that bacilli, indeed, improve the abundance and activity of lactobacilli [107,108]. This was attributed to the synthesis of heme by bacilli, which is necessary for the heme-dependent catalysis of lactobacilli [107]. However, it seems that there might be another mechanism—providing lactobacilli with prebiotic sugars that are difficult for others to access, i.e., energy. In our prior works [109], we have shown that the probiotic activity of lactobacilli strongly depends on the availability of available sugars in the medium. It is possible that the provision of fructo-oligosaccharides to lactobacilli also contributes to a more active production of lactic acid and metabolites by lactobacilli, improving their probiotic activity.

Notably, our analysis revealed no unexpected absences or exceptions in the correlation between phenotypic screening and genomic confirmation, which confirms the validity of our screening approach.

### 4.3. Effect on Fish Growth and Feed Conversion

Accelerated somatic growth is extremely significant for fish during early ontogenesis, as it increases the individual competitiveness and survival rates after wintering [110]. The use of probiotics stabilizes intestinal microbiota composition, which improves the activity of digestive enzymes and, as a result, feed digestibility [111].

In aquaculture species, *B. velezensis* was shown to induce improved growth. Hybrid mudcat showed better growth rates when fed diets containing *B. velezensis* YFI-E109 [112]. Pacific white shrimp showed an increase in final body length, weight gain rate, and specific growth rate when fed diets supplemented with *B. velezensis* BV007 [113]. Similar growth stimulation effects were observed in carp [114] and grouper [115].

It aligns with observed effects of our *B. velezensis* strains. Our two groups of probiotics induced a 31.9–42.1% increase in individual biomass growth and a 5.04–6.47% reduction in feed conversion ratio (FCR). Notably, we observed reduction in feed conversion rate in experimental groups. In terms of industrial production, this corresponds to a saving of 6.47 kg of feed per 100 kg of live weight gain. For a fish farm with an annual production volume of 100 tons of carp, the use of a probiotic preparation can reduce feed consumption by 12.94 tons. With an average market price of carp feed compound of USD1.2/kg, the potential annual savings will be about USD15,528. The results obtained demonstrate the practical value of the study for improving the economic efficiency of commercial carp farming.

In comparison, hybrid yellow catfish (*Pelteobagrus* spp.) fed with *B. velezensis* YFI-E109 showed an 18% weight gain increase and 15.8% FCR reduction [112]. In a study [115] *B. velezensis* induced a 15.0% increase in weight gain rate compared to control.

The observed growth improvement seems to be directly related to the activity of the probiotics used: additional lytic enzymes produced by bacilli improved the overall digestibility and the breakdown of complex feed components, allowing fish to extract more energy and nutrients per unit of feed than the control group. As stated above, our genomic and functional analyses of new strains confirmed the presence of genes for key digestive enzymes and stress resistance, supporting the hypothesis that these strains act mainly by improving digestion rather than directly modulating host gene expression.

It should be noted, though, that the observed growth improvements substantially exceed typical *Bacillus velezensis* effects reported in literature, which validates our screening approach. However, the FCR reductions fall within the lower range of reported improvements, while some studies achieve 15–20% FCR reductions.

The observed pattern seems to reflect the distinct underlying mechanisms that operate independently in aquaculture probiotic supplementation. Growth promotion and feed conversion efficiency could represent distinct probiotic mechanisms with different optimization requirements. Our new strains likely enhanced growth performance through enzymatic activity and nutrient absorption rather than feed efficiency optimization.

A meta-analysis [116] that examined the effects of probiotics on specific growth rate (SGR) and feed conversion ratio (FCR) in commercial fish species revealed that the minimum effective concentration of probiotics for improving SGR was 10^7^ log CFU/g, while 10^8^ log CFU/g was needed to enhance FCR. A supplementation at 0.1% (approximately 10^6^–10^7^ CFU/g) may have been optimal for growth but suboptimal for maximizing feed conversion efficiency.

Since in our experimental setup no force-feeding methods were employed and all consumption was based on the fish’s natural feeding behavior, one possible interpretation for our results is that the *Bacillus velezensis* MT strains enhanced voluntary feed intake through appetite. It is known that probiotics can influence the gut–brain axis by modulating the expression of key appetite-regulating neuropeptides [117], and also can reduce stress levels, which is associated with improved appetite and feeding behavior [118]. These assumptions open up some new promising directions for future research. In addition, a promising area of further research is to evaluate the effectiveness of the probiotic strains under study when they are included in compound feeds with a higher content of plant components.

### 4.4. Colonization and Stability of Preparations In Vivo

One of the important issues in probiotics efficacy is the survival of probiotic bacteria within the host body. Carps lack a developed stomach with acidic conditions, eliminating concerns about probiotic viability in a low-pH environment. However, we investigated whether sediment-derived *Bacillus* spores could germinate and proliferate in the intestinal lumen.

As can be seen from Table 10, the number of probiotic strains in the intestinal contents was comparable to the amount obtained with feed (10^6^ CFU/g). That is, the amount of probiotic in the intestinal contents did not increase, but the vast majority of the population was in a vegetative state.

In the control group, the carp intestine contained only 10^4^ CFU/g of bacilli, which is significantly less than the amount introduced with the feed and observed in the intestines of the experimental groups. It is likely that the suppression of the growth of the probiotic bacilli population, even considering their vegetative state, is related to their excess in the intestine. It should be noted that some studies have even reported a decrease in the number of probiotic bacilli in the intestine relative to their content in the feed [105,119]. We can assume that this suppression is due both to the action of the rest of the intestinal microbiota of the carp and to the host organism’s response, but these assumptions require further verification in future studies.

### 4.5. Secondary Metabolites and Antimicrobial Activity

The ability to synthesize non-ribosomal peptides is a relatively new and rarely discussed feature of probiotics. However, accumulating evidence suggests that NRP may significantly contribute to the effects of probiotic *Bacillus* strains due to their antimicrobial, immunomodulatory, and intestinal health-enhancing properties.

The genomic analysis of four new strains revealed several clusters of genes for non-ribosomal synthesis of bacilysin, difficidin, macrolactin, bacillaene, mycosubtilin, surfactin, and bacillomycin. Bacilysin, surfactin, macrolactin H, and bacillaene were found in all four strains.

This class of substances is primarily known for antimicrobial and antifungal effects, but some representatives have other properties, for example, antiviral, anti-cancer, immunomodulatory, antioxidant, and quorum quenching against pathogens [120,121,122]. The main effects of the relevant substances are shown in Table 7.

Notably, only bacilysin and bacillaene had 100% similarity to reference peptides in the AntiSMASH database, while for other substances there were deviations from 100% identity. This suggests that these strains are capable of producing modified variants of non-ribosomal peptides, such as different isoforms of surfactin, which can provide them with a broader spectrum of activity. Such variability in NRP structure allows producing strains to better adapt to different antagonists. It has been shown that a *Bacillus* strain producing only one isoform of surfactin is less effective than a strain producing several isoforms [123].

Bacillysin is a peptide with antimicrobial activity: it inhibits glucosamine synthetase, disrupting peptidoglycan synthesis and causing cell lysis in pathogens. It suppresses genes involved in pathogen virulence and cell wall biosynthesis [124] and is effective against many gram-negative pathogens [125].

Bacillaene is a molecule of internal signaling in the bacterial cell. In low concentrations (0.1–1.0 micrograms/mL), it accelerates the formation of biofilm in *Bacillus*, and in higher concentrations it suppresses competing microbes due to structural instability, forming decomposition products that destroy pathogens [126].

The combined action of these two compounds can create a selective advantage for our introduced strains, explaining the microbiological data showing that spore-to-vegetative cell conversion rates reached 94–95% while displacing native bacilli. Their selective antimicrobial activity, rather than broad-spectrum inhibition, explains why the overall microbial diversity remained largely unchanged despite the significant shift in *Bacillus* populations.

Difficidin is a broad-spectrum antibiotic effective against gram-negative bacteria [124,127]. Macrolactin is antibacterial and anti-inflammatory substance; it suppresses the activity of bacterial ATPase and reduces oxidative stress in host cells. It is also known to contribute to the modulation of the microbiota [128]. Mycosubtilin and bacillomycin are primarily antifungal agents [82,129]. In *Bacillus*, bacillomycin also enhances biofilm formation and increases the binding of siderophores [130] and acts against several pathogens [131]. Surfactin is a multifunctional compound antagonistic to bacteria, fungi, and viruses, that has an anti-inflammatory effect and recognized as a biomarker of probiotic activity, as high surfactin production correlates with the effectiveness of probiotics in colonization of the intestine [132].

Based on our data, we believe that bacillisin and bacillaene may also be markers of probiotic activity in *Bacillus* strains alongside surfactin.

### 4.6. Gene Expression Analysis and Mechanisms of Probiotic Action

Bacterial metabolites can also directly or indirectly affect gene expression. For example, we previously demonstrated that probiotic bacilli are able to modulate gene expression in the clariid catfish [133]. There is also evidence that *B. velezensis* probiotics affect gene expression in fish and activate genes associated with immunity: IgM (increase in 2.5 times in the spleen), TNF-α (increase in 3 times in the kidneys), MHCI and IgD in lymphoid organs, as well as modulate the expression of antioxidant genes: superoxide dismutase (SOD) and catalase (CAT) [104,114].

All of these effects correlate with increased survival and other positive phenotypic effects in hosts [76].

Guided by the hypothesis that our probiotics may have a similar effect, we included measurement of gene expression in the design of this study. However, no significant transcriptional changes were observed. Apparently, these strains can be attributed to “direct-acting” probiotics rather than to “regulatory” ones, and their effect is ensured by optimizing digestion due to enzyme activity and antimicrobial action, including through the activity of non-ribosomally synthesized peptides. This conclusion is further supported by the observed reduction in feed conversion ratio.

The effects of probiotics are inherently strain-specific [104]. Therefore, it is especially important to monitor the effects and mechanisms of action of individual strains.

This may be due to the fact that for aquaculture species, bacilli are a transient (allochthonous) microbiota that does not remain in the fish intestine for more than a few days after the start of feeding [134,135,136]. Therefore, unlike autochthonous microbiota, they have not developed a unified strategy for symbiotic interaction with fish hosts, and when they enter the intestinal microbial community, they demonstrate flexibility by choosing one of the many strategies.

### 4.7. Limitations and Future Directions

Several important limitations should be acknowledged in our experimental design and methodology.

While the study provides compelling evidence for the growth-promoting effects of these probiotics, the gene expression analysis may have been limited to a selected set of muscle genes and may not capture broader immunological or metabolic changes. We conclude that these four *Bacillus velezensis* strains are “direct-acting” rather than “regulatory” probiotics based on the absence of significant transcriptional changes in muscle tissue, but this interpretation must be considered within the context of our experimental design limitations.

The choice of muscle tissue for gene expression analysis, while practically convenient and commonly used in aquaculture research [137], may not represent all probiotic-mediated regulatory effects. Recent studies have demonstrated that some probiotics influence gene expression in the gastrointestinal tract of fish [138], but that modulation of genes involved in immune response, metabolic pathways, and stress regulation sometimes are not detectable in peripheral tissues [139].

Additionally, examining gene expression in other immunologically relevant tissues such as spleen, head kidney, and liver would provide a more complete understanding of whether these strains truly lack regulatory properties or whether our current methodology simply failed to detect tissue-specific and temporally dynamic transcriptional responses.

Furthermore, the temporal dynamics of probiotic effects may vary significantly between tissues, with gut-associated changes potentially occurring earlier or later than those in muscle tissue. The timing of our sampling could have missed critical windows of gene expression modulation, as studies have shown that probiotic-induced transcriptional changes can be highly time-dependent, with some effects manifesting within days of administration while others require weeks to become apparent [118].

Another limitation that should be taken into account is that our experimental design utilized a single tank per treatment group. This design, while presenting statistical considerations regarding pseudoreplication, follows established practices in aquaculture research, where resource constraints and practical considerations necessitate this approach. For example, in work [140] to maintain appropriate production densities of fish, experiments were conducted in one tank per diet until day 86. A similar design has been successfully employed in aquaculture studies, including research on feeding rates, probiotic supplementation in various fish species, and growth performance evaluations [141,142].

Studies have demonstrated that while tank effects can influence results, this experimental approach remains valid when environmental conditions are carefully controlled and monitored, as was maintained throughout our 76-day trial [143]. Many successful aquaculture nutrition studies have utilized similar single-tank-per-treatment designs while acknowledging the statistical implications. Recent surveys of aquaculture growth studies indicate that approximately 80% of published research applies treatments in limited replication scenarios, with researchers recognizing that practical constraints often necessitate balancing statistical power with available resources [144]. This approach helps us to focus on demonstrating biological efficacy, particularly in preliminary screening studies.

The other possible limitation—the absence of quantitative metabolite analysis in our study—reflects a common limitation in probiotic research, where genomic potential identification often precedes comprehensive metabolomic validation [145]. Current research in aquaculture probiotics frequently relies on genomic screening to identify bioactive compound potential, with metabolite quantification representing a subsequent research phase that requires specialized analytical equipment and methodologies [146].

The correlation between genetic potential and actual in vivo production levels can vary considerably in different environmental conditions, host factors, and bacterial growth phases [147]. This identifies another possible future research direction—studying the expression of these genes in vivo. Our genomic identification of key metabolite biosynthetic pathways provides a solid foundation for future metabolomic studies, following the established research progression from genetic screening to functional validation that characterizes the field of aquaculture probiotic development.

The gap between genomic predictions and metabolite quantification is acknowledged as a current frontier in probiotic research, with studies increasingly implementing both approaches to provide better understanding of probiotic mechanisms.

Therefore, we believe that future research on these strains should prioritize transcriptomic analysis of gut mucosa and other tissue samples collected at multiple time points to capture the full spectrum of potential regulatory mechanisms. We would also like to suggest focusing on elucidating additional mechanisms of probiotics action, including regulatory interactions with the gut microbiota and host metabolism. Metabolomic profiling of lipopeptides and other secondary metabolites in intestinal contents should be implemented using advanced analytical platforms such as mass spectrometry to quantify the actual production levels of bioactive compounds identified through genomic analysis.

Multi-location validation trials across different aquaculture facilities would address the generalizability of our findings and account for environmental variability that could influence probiotic efficacy. Additional research directions should include longitudinal studies of gut microbiota dynamics using 16S rRNA sequencing combined with host transcriptomics to understand the temporal establishment of probiotic strains and their impact on indigenous microbial communities.

Finally, the development of standardized protocols for probiotic delivery, dosage optimization, and effect monitoring would facilitate the translation of these research findings into practical aquaculture applications.

### 4.8. Practical Implications for Sustainable Aquaculture

Despite the acknowledged limitations in our experimental design and methodology, we believe that this study has successfully accomplished its primary objectives, establishing a comprehensive pipeline for the initial screening of novel probiotic strains and demonstrating the correlation between genomic predictions and in vivo performance. Most importantly, our in vivo trials demonstrated that the genomic potential identified through bioinformatics analysis translated into measurable biological effects. This work also provides validated genetic markers that can guide the selection of similar strains for aquaculture.

The demonstrated efficacy of these *Bacillus* strains in enhancing growth on plant-based feeds supports their application as sustainable alternatives to antibiotics in aquaculture. The integration of genomic screening in probiotic development, as exemplified in this study, offers a powerful approach for identifying strains with optimal traits for specific aquaculture challenges.

Potentially, these probiotic strains could also offer substantial environmental advantages by improving water quality through enhanced breakdown of organic matter and reduced nutrient loading in aquaculture effluents.

The enzymatic capabilities of the characterized strains, particularly their cellulolytic and proteolytic activities, directly address aquaculture’s transition toward plant-based feed systems by improving digestibility of complex plant materials and reducing dependence on fishmeal-based feeds. This enzymatic enhancement makes plant-based diets more economically viable while supporting sustainable feed formulations using locally available materials.

The integration of probiotic supplementation provides significant cost reductions through improved feed conversion ratios, reduced veterinary treatment expenses, and enhanced survival rates. The stable spore-forming nature of *Bacillus* species ensures longer shelf life and easier storage, improving commercial viability. Additionally, these probiotics support global efforts to reduce antibiotic use in aquaculture, enhancing regulatory compliance and market access for aquaculture products, particularly in markets with strict antibiotic residue standards.

The practical implementation is facilitated by scalable solid-phase fermentation using readily available substrates like soybeans, making the technology accessible and cost-effective.

## 5. Conclusions

In this study, four new strains of *Bacillus velezensis* were characterized as potential probiotics for aquaculture. These strains were isolated from river sediments and selected based on their high enzymatic activity and antibiotic sensitivity profile. Probiotic formulations were developed based on compatibility testing and included two pairs of probiotics that were able to form joint biofilms. In vivo experiments on carp have shown that probiotics supplementation increases fish weight gain and reduces feed conversion ratio, with MT14 and MT42 strains combination yielding the highest average weight achieved.

Functional genomic analysis of the revealed key genes responsible for probiotic activity, including those conferring resistance to various types of stress and synthesis of bioactive metabolites. The results also indicate that the mechanism of action of these probiotics is primarily mediated by their enzymatic activity.

Beyond characterizing novel aquaculture probiotics, this work identifies genetic markers for the selection of similar strains in the future. These genetic markers and pipeline can guide the rational design of multi-strain probiotics tailored to specific aquaculture systems, ultimately reducing reliance on fishmeal and antibiotics.

## Figures and Tables

**Table 1:** Primers for studying the expression of stress and immunity genes in carp.

Target Gene	Forward Primer Sequence (F, 5′-3′)	Reverse Primer Sequence (R, 5′-3′)	Product Length	Melting Point of Primers, °C	GC Content, %
*act*	GTCTACCACTTCGCCCTCATC	CAGTGTACAGAGACACCCTGG	247	F: 60.20 R: 59.73	F:57.14 R:57.14
*cxc*	CTGGGATTCCTGACCATTGGT	GTTGGCTCTCTGTTTCAATGCA	88	F: 55.87 R: 57.46	F: 52 R: 45
*lyz*	GTGTCTGATGTGGCTGTGCT	TTCCCCAGGTATCCCATGAT	359	F: 60.38 R: 57.46	F: 55 R: 50
*hsp70*	TGAGAACATCAACGAGCCCA	TTGTCAAAGTCCTCCCCACC	195	F: 59.59 R: 59.62	F: 50 R: 55
*il-1β*	ACTGGAGCTGTCTTCGCATC	CTCCAAGATGAAGCCGAGCA	136	F: 58.48 R: 59.25	F: 55 R: 55
*il-10*	GCTGTCACGTCATGAACGAGAT	CCCGCTTGAGATCCTGAAATAT	132	F: 58.89 R: 57.01	F: 50 R: 45
*igf-1*	CCGTCTCCTGTTCGCTAAATCT	CTTTGGTGTCCTGGGACTGT	554	F: 60.86 R: 59.02	F: 50 R: 55
*gst*	TACAATACTTTCACGCTTTCCC	GGCTCAACACCTCCTTCAC	149	F: 54.41 R: 54.25	F: 41 R: 58
*β2m*	CCAAATACCCAGCAGACGGA	CAGTTGCTAGGCAGACGTTTA	784	F: 56.75 R: 57.2	F: 55 R: 48
*mt1*	ATGGATCCTTGCGATTGCGCCA	CGAACAGGTTCACATAGGTGA	232	F: 62.94 R: 55.04	F: 55 R: 48

**Table 2:** Values of the width of the clearance zone around strains grown on the milk medium (proteolytic activity) and starch medium (amylolytic activity).

Strain	Proteolytic Activity (Width of the Clearing Zone in the Milk Medium, mm)	Amylolytic Activity (Width of the Clearing Zone in the Starch Medium, mm)	Cellulolytic Activity (Width of the Clearing Zone in the Carboxymethilcellulose Medium, mm)
MT14	8 ± 0.3	8 ± 0.6	16 ± 0.3
MT27	8 ± 0.6	8 ± 1.0	-
MT42	8 ± 0.3	11 ± 1.0	17 ± 1.0
MT48	6 ± 1.0	11 ± 0.3	16 ± 0.3
MT49	6 ± 0.6	10 ± 1.0	-
MT56	5 ± 1.0	10 ± 0.3	-
MT64	9 ± 0.3	4 ± 1.0	12 ± 0.6
MT73	5 ± 0.6	10 ± 0.3	-
MT74	8 ± 1.0	8 ± 1.0	16 ± 1.0
MT77	3 ± 0.6	10 ± 0.3	6 ± 0.3
MT84	9 ± 0.3	2 ± 0.6	-
MT102	10 ± 0.6	4 ± 1.0	18 ± 1.0
MT141	11 ± 0.3	6 ± 0.3	14 ± 0.3
MT142	10 ± 1.0	5 ± 1.0	15 ± 0.6
B1895a	5 ± 0.3	7 ± 0.6	-
Katmira	5 ± 1.0	4 ± 0.3	-

**Table 3:** The interaction among strains.

	MT14	MT27	MT42	MT48	MT49	MT56	MT64	MT73	MT74	MT77	MT84	MT102	MT141	MT142
MT14	%	=	+	+	=	=	+	=	−	−	−	−	+	+
MT27	=	%	=	=	=	=	=	=	−	−	=	−	=	=
MT42	+	=	%	=	=	=	=	=	=	=	=	=	+	=
MT48	+	=	=	%	=	=	=	=	=	=	−	=	+	=
MT49	=	=	=	=	%	=	=	=	=	=	=	−	=	=
MT56	=	=	=	=	=	%	=	=	=	=	=	=	=	+
MT64	+	=	=	=	=	=	%	+	=	−	−	−	=	=
MT73	=	=	=	=	=	=	+	%	−	=	−	=	+	=
MT74	−	−	=	=	=	=	=	−	%	−	−	=	−	=
MT77	−	−	=	=	=	=	−	=	−	%	−	−	−	−
MT84	−	=	=	−	=	=	−	−	−	−	%	−	−	=
MT102	−	−	=	=	−	=	−	=	=	−	−	%	−	+
MT141	+	=	+	+	=	=	=	+	−	−	−	−	%	+
MT142	+	=	=	=	=	+	=	=	=	−	=	+	+	%

Signs “+”, “−“, and “=” marks interaction types described above. The % sign marks self-comparison.

**Table 4:** Assembly statistics and genome features of *B. velezensis* strains.

Strain	MT14	MT42	MT141	MT142
Length b.p.	4,080,572	4,115,985	4,432,692	4,434,607
Contigs count	6	3	3	3
GC %	46.2	46.1	45.4	45.4
N50 b.p.	1,035,922	4,080,892	4,303,216	4,305,438
Coding density %	88.8	89.4	88.8	88.9
tRNAs	87	92	97	93
tmRNAs	1	1	1	1
rRNAs	26	30	31	31
ncRNAs	31	31	34	34
ncRNA regions	58	58	58	58
CRISPR arrays	0	0	0	0
CDSs	4571	4433	4918	4813
pseudogenes	343	245	280	215
hypotheticals proteins	515	388	605	522
signal peptides	0	0	0	0
sORFs	3	4	5	5
gaps	0	0	0	0
oriCs	2	2	2	2
Assembly completeness %	97.35	99.97	99.94	99.91
Assembly contamination %	2.19	2.1	1.19	2.32

**Table 5:** Lytic enzymes of the studied strains. The number of “+” is the number of detected copies of genes.

Enzyme	MT14	MT42	MT141	MT142
Glycosyl hydrolases of the alpha-amylase family	+++	+	+++	+
Alpha-amylase	++	++	+	+
Glucosidase/amylase (phosphorylase)	+	+	+	
Beta-glucanase	+	+	+	+
6-phosphorus-beta-glucosidase	++++	++	++	++++
6-beta-D-glucan-glucanohydrolase	+	++	++	+
Alpha-D-1,4-glucosidase	+	+		
Oligo-1,6-glucosidase				+
Aryl-phosphorus-beta-D-glucosidase BglC	+	+	++	+
Aryl-phosphorus-beta-D-glucosidase BglH			+	
Maltodextrin-glucosidase	+	+		+
Maltose-6′-phosphate glucosidase	+	+	+++	+
Chitobiose-specific 6-phosphorus-beta-glucosidase ChbF	+	++	+	
Neopullalanase	+	+	+	
Levansaccharase	+	+	++	++
Alpha, alpha-phosphotregalase			+	+
DNA-recombinase SpoIVCA/DNA-invertase PinE			+	+
DNA-invertase hin		+		+
Cellulose		+		+
Endoglucanase	+	+	++	+
Protein of the lysozyme family	+	+	+	
CwlT-like protein containing lysozyme domain	+	+	+	++
Hemolysin C	+		+	
Hemolysin III family protein	+	+	+	+
Hemolysins and related proteins containing CBS domains		+++		+

**Table 6:** Resistance genes clusters.

Drug Resistance Cluster	Strain			
	14	42	141	142
M00627 beta-Lactam resistance, Bla system (1)	(2 blocks missing 1/3)	(2 blocks missing 1/3)	(2 blocks missing 1/3)	(2 blocks missing 1/3)
M00704 Tetracycline resistance, efflux pump Tet38 (1)	(1 block missing 1/2)	(1 block missing 1/2)	(1 block missing 1/2)	(1 block missing 1/2)
M00726 Cationic antimicrobial peptide (CAMP) resistance, lysyl-phosphatidylglycerol (L-PG) synthase MprF (1)	(2 blocks missing 1/3)	(2 blocks missing 1/3)	(2 blocks missing 1/3)	(2 blocks missing 1/3)
M00700 Efflux pump AbcA (2)	(complete 2/2)	(complete 2/2)	(complete 2/2)	(complete 2/2)
M00702 Multidrug resistance, efflux pump NorB (1)	(1 block missing 1/2)	(1 block missing 1/2)	(1 block missing 1/2)	(1 block missing 1/2)
M00714 Multidrug resistance, efflux pump QacA (1)	(1 block missing 1/2)	(1 block missing 1/2)	(1 block missing 1/2)	(1 block missing 1/2)

**Table 7:** Genes for the synthesis of secondary metabolites found in the analyzed genomes.

Substance/Strain	*B. velezensis* MT14	*B. velezensis* MT42	*B. velezensis* MT141	*B. velezensis* MT142	Possible Activity
Bacilysin	100	100	100	100	Antimicrobial [76]
Surfactin	78	78	78	78	Antimicrobial, immunostimulation [77,78]
Microlactin (H)	88	100	90	90	Antiviral [79]
Bacillaene	100	100	100	71	Antibacterial [79]
Mycosubtilin	100	-	100	-	Antifungal [80]
Difficidin	8	100	100	93	Antibacterial [81]
Bacillomycin	-	100	-	100	Antifungal [82]

**Table 8:** Size and weight characteristics of juvenile carp *Cyprinus carpio* Linnaeus, 1758 when adding multistrain probiotic feed additives (0.1%) based on four new strains of *Bacillus velezensis* to the diet.

Parameter	Control Group		Experimental Group No. 1 MT14 + MT42		Experimental Group No. 2 MT141 + MT142	
	Day 1	Day 76	Day 1	Day 76	Day 1	Day 76
Individual weight (g)						
min	1.65	6.51	1.78	10.54	1.37	7.82
max	6.69	32.87	6.42	44.71	5.69	45.63
Average value ± SD	3.79 ± 0.96	17.97 ± 3.73	3.67 ± 0.86	23.82 ± 6.23	2.91 ± 0.72	21.61 ± 7.11
Individual length (cm)						
min	4.26	8.91	4.19	9.51	3.64	7.48
max	6.51	12.46	6.32	13.49	5.78	13.10
Average value ± SD	5.05 ± 0.44	10.41 ± 0.73	5.02 ± 0.41	11.32 ± 0.73	4.51 ± 0.36	10.61 ± 0.99
Fulton’s fatness coefficient, standard units	2.94	1.59	2.91	1.64	3.17	1.81

**Table 9:** Fish-breeding indices of juvenile carp *Cyprinus carpio* Linnaeus, 1758 when added to the diet of multi-strain probiotic feed additives (0.1%) based on four new strains of *Bacillus velezensis*.

Criteria	Control Group	Experimental Group No. 1 MT14 + MT42	Experimental Group No. 2 MT141 + MT142
Fish biomass, kg (during the end of the experiment)	2.55	3.48	3.07
Total increase in biomass, kg	1.98	2.93	2.63
Biomass growth, %	448.85	631.74	703.00
Individual biomass growth, g	14.18	20.15	18.7
Individual biomass growth, %	474.14	649.05	742.61
Specific mass growth rate, %/day	2.04	2.47	2.63
Specific growth rate of fish by length, %/day	0.95	1.08	1.12
Feed conversion rate, kg/kg	1.39	1.32	1.30
Survival rate, %	94.67	97.33	94.67

**Table 10:** The content of bacteria morphologically similar to the corresponding probiotics in the intestinal contents of carp fingerlings of different ages, CFU/g.

Probiotic Used	Total Amount	Spore Form
Control	3.2 ± 0.2·10^4^	5.3 ± 0.4·10^3^
Experiment 1 (MT14 + MT42)	2.7 ± 0.4·10^6^	1.5 ± 0.2·10^4^
Experiment 2 (MT141 + MT142)	2.3 ± 0.3·10^6^	1.1 ± 0.4·10^4^

## Data Availability

The original genomic data presented in the study are openly available in the NCBI database under accession numbers JBOZKB000000000 (MT14), JBOZKA000000000 (MT42), JBPIYB000000000 (MT141), and JBPIYC000000000 (MT142), or through BioProject PRJNA1269758. Other experimental data are available upon request.

## References

[1] Food And Agriculture Organization (FAO) (2018). The State of World Fisheries and Aquaculture 2018 (SOFIA).

[2] Food And Agriculture Organization (FAO) (2020). The State of World Fisheries and Aquaculture 2020.

[3] El-Saadony M.T., Alagawany M., Patra A.K., Kar I., Tiwari R., Dawood M.A.O., Dhama K., Abdel-Latif H.M.R. (2021). The Functionality of Probiotics in Aquaculture: An Overview. Fish Shellfish Immunol..

[4] Naylor R.L., Kishore A., Sumaila U.R., Issifu I., Hunter B.P., Belton B., Bush S.R., Cao L., Gelcich S., Gephart J.A. (2021). Blue Food Demand across Geographic and Temporal Scales. Nat. Commun..

[5] Kroeckel S., Harjes A.-G.E., Roth I., Katz H., Wuertz S., Susenbeth A., Schulz C. (2012). When a Turbot Catches a Fly: Evaluation of a Pre-Pupae Meal of the Black Soldier Fly (*Hermetia illucens*) as Fish Meal Substitute—Growth Performance and Chitin Degradation in Juvenile Turbot (*Psetta maxima*). Aquaculture.

[6] Zhou C., Xu D., Lin K., Sun C., Yang X. (2018). Intelligent Feeding Control Methods in Aquaculture with an Emphasis on Fish: A Review. Rev. Aquac..

[7] Eljasik P., Panicz R., Sobczak M., Sadowski J. (2022). Key Performance Indicators of Common Carp (*Cyprinus carpio* L.) Wintering in a Pond and RAS under Different Feeding Schemes. Sustainability.

[8] Nasr M.A.F., Reda R.M., Ismail T.A., Moustafa A. (2021). Growth, Hemato-Biochemical Parameters, Body Composition, and Myostatin Gene Expression of *Clarias gariepinus* Fed by Replacing Fishmeal with Plant Protein. Animals.

[9] Zhao W., Liu Z.-L., Niu J. (2021). Growth Performance, Intestinal Histomorphology, Body Composition, Hematological and Antioxidant Parameters of *Oncorhynchus mykiss* Were Not Detrimentally Affected by Replacement of Fish Meal with Concentrated Dephenolization Cottonseed Protein. Aquac. Rep..

[10] Tusche K., Nagel F., Arning S., Wuertz S., Susenbeth A., Schulz C. (2013). Effect of Different Dietary Levels of Potato Protein Concentrate Supplemented with Feed Attractants on Growth Performance of Rainbow Trout (*Oncorhynchus mykiss*). Anim. Feed. Sci. Technol..

[11] Manhar A.K., Bashir Y., Saikia D., Nath D., Gupta K., Konwar B.K., Mandal M. (2016). Cellulolytic potential of probiotic *Bacillus subtilis* AMS6 isolated from traditional fermented soybean (Churpi): An in-vitro study with regards to application as an animal feed additive. Microbiol. Res..

[12] Wuertz S., Schroeder A., Wanka K.M. (2021). Probiotics in fish nutrition—Long-standing household remedy or native nutraceuticals?. Water.

[13] Maity J., Kundu J., Pramanik A., Patra B.C. (2011). Effect of cellulolytic gut bacteria as a feed supplement on the growth performance and nutrient digestibility of Asian seabass (*Lates calcarifer*). Int. J. Aquat. Sci..

[14] Ibrahem M.D. (2015). Evolution of Probiotics in Aquatic World: Potential Effects, the Current Status in Egypt and Recent Prospectives. J. Adv. Res..

[15] Kuebutornye F.K.A., Abarike E.D., Lu Y. (2019). A Review on the Application of Bacillus as Probiotics in Aquaculture. Fish Shellfish Immunol..

[16] Hill C., Guarner F., Reid G., Gibson G.R., Merenstein D.J., Pot B., Morelli L., Canani R.B., Flint H.J., Salminen S. (2014). The International Scientific Association for Probiotics and Prebiotics Consensus Statement on the Scope and Appropriate Use of the Term Probiotic. Nat. Rev. Gastroenterol. Hepatol..

[17] Monzón-Atienza L., Bravo J., Serradell A., Montero D., Gómez-Mercader A., Acosta F. (2023). Current Status of Probiotics in European Sea Bass Aquaculture as One Important Mediterranean and Atlantic Commercial Species: A Review. Animals.

[18] Monzón-Atienza L., Bravo J., Torrecillas S., Gómez-Mercader A., Montero D., Ramos-Vivas J., Galindo-Villegas J., Acosta F. (2024). An In-Depth Study on the Inhibition of Quorum Sensing by *Bacillus velezensis* D-18: Its Significant Impact on Vibrio Biofilm Formation in Aquaculture. Microorganisms.

[19] Kuebutornye F.K.A., Abarike E.D., Lu Y., Hlordzi V., Sakyi M.E., Afriyie G., Wang Z., Li Y., Xie C.X. (2020). Mechanisms and the Role of Probiotic *Bacillus* in Mitigating Fish Pathogens in Aquaculture. Fish Physiol. Biochem..

[20] Zhou X., Wang Y., Li W. (2009). Effect of Probiotic on Larvae Shrimp (*Penaeus vannamei*) Based on Water Quality, Survival Rate and Digestive Enzyme Activities. Aquaculture.

[21] Liu H., Wang S., Cai Y., Guo X., Cao Z., Zhang Y., Liu S., Yuan W., Zhu W., Zheng Y. (2017). Dietary Administration of Bacillus Subtilis HAINUP40 Enhances Growth, Digestive Enzyme Activities, Innate Immune Responses and Disease Resistance of Tilapia, *Oreochromis niloticus*. Fish Shellfish Immunol..

[22] Santos R.A., Oliva-Teles A., Pousão-Ferreira P., Jerusik R., Saavedra M.J., Enes P., Serra C.R. (2021). Isolation and Characterization of Fish-Gut *Bacillus* spp. as Source of Natural Antimicrobial Compounds to Fight Aquaculture Bacterial Diseases. Mar. Biotechnol..

[23] Sankar H., Philip B., Philip R., Singh I.S.B. (2017). Effect of Probiotics on Digestive Enzyme Activities and Growth of Cichlids, *Etroplus suratensis* (Pearl Spot) and *Oreochromis mossambicus* (Tilapia). Aquacult. Nutr..

[24] Adorian T.J., Jamali H., Farsani H.G., Darvishi P., Hasanpour S., Bagheri T., Roozbehfar R. (2019). Effects of Probiotic Bacteria *Bacillus* on Growth Performance, Digestive Enzyme Activity, and Hematological Parameters of Asian Sea Bass, *Lates calcarifer* (Bloch). Probiotics Antimicrob. Proteins.

[25] Sumon M.S., Ahmmed F., Khushi S.S., Ahmmed M.K., Rouf M.A., Chisty A.H., Sarower G. (2018). Growth Performance, Digestive Enzyme Activity and Immune Response of *Macrobrachium rosenbergii* Fed with Probiotic *Clostridium butyricum* Incorporated Diets. J. King Saud Univ. Sci..

[26] Afrilasari W., Widanarni, Meryandini A. (2016). Effect of Probiotic *Bacillus megaterium* PTB 1.4 on the Population of Intestinal Microflora, Digestive Enzyme Activity and the Growth of Catfish (*Clarias* sp.). HAYATI J. Biosci..

[27] Ringø E., Zhou Z., Vecino J.L.G., Wadsworth S., Romero J., Krogdahl A., Olsen R.E., Dimitroglou A., Foey A., Davies S. (2016). Effect of Dietary Components on the Gut Microbiota of Aquatic Animals. A Never-Ending Story?. Aquac. Nutr..

[28] Martínez Cruz P., Ibáñez A.L., Monroy Hermosillo O.A., Ramírez Saad H.C. (2012). Use of Probiotics in Aquaculture. ISRN Microbiol..

[29] Liu X.-F., Li Y., Li J.-R., Cai L.-Y., Li X.-X., Chen J.-R., Lyu S.-X. (2015). Isolation and Characterisation of *Bacillus* spp. Antagonistic to *Vibrio parahaemolyticus* for Use as Probiotics in Aquaculture. World J. Microbiol. Biotechnol..

[30] Meidong R., Khotchanalekha K., Doolgindachbaporn S., Nagasawa T., Nakao M., Sakai K., Tongpim S. (2018). Evaluation of Probiotic *Bacillus aerius* B81e Isolated from Healthy Hybrid Catfish on Growth, Disease Resistance and Innate Immunity of Pla-Mong *Pangasius bocourti*. Fish Shellfish Immunol..

[31] Lee S., Katya K., Park Y., Won S., Seong M., Hamidoghli A., Bai S.C. (2017). Comparative Evaluation of Dietary Probiotics *Bacillus subtilis* WB60 and *Lactobacillus plantarum* KCTC3928 on the Growth Performance, Immunological Parameters, Gut Morphology and Disease Resistance in Japanese Eel, *Anguilla japonica*. Fish Shellfish Immunol..

[32] Niu K.-M., Kothari D., Lee W.-D., Lim J.-M., Khosravi S., Lee S.-M., Lee B.-J., Kim K.-W., Han H.-S., Kim S.-K. (2019). Autochthonous *Bacillus licheniformis*: Probiotic Potential and Survival Ability in Low-Fishmeal Extruded Pellet Aquafeed. Microbiologyopen.

[33] Yaylacı E.U. (2021). Isolation and Characterization of *Bacillus* spp. from Aquaculture Cage Water and Its Inhibitory Effect against Selected *Vibrio* spp. Arch. Microbiol..

[34] Nakharuthai C., Boonanuntanasarn S., Kaewda J., Manassila P. (2023). Isolation of Potential Probiotic *Bacillus* spp. from the Intestine of Nile Tilapia to Construct Recombinant Probiotic Expressing CC Chemokine and Its Effectiveness on Innate Immune Responses in Nile Tilapia. Animals.

[35] Elmnasser N., Hassen W., Zmantar T., Ashraf S.A., Hadj Lajimi R., Humaidi J.R., Alreshidi M., Hamadou W.S., Emira N., Snoussi M. (2024). Antagonistic and Enzymatic Activities of Bacillus Species Isolated from the Fish Gastrointestinal Tract as Potential Probiotics Use in Artemia Culture. Cell. Mol. Biol..

[36] Tan H.Y., Chen S.-W., Hu S.-Y. (2019). Improvements in the Growth Performance, Immunity, Disease Resistance, and Gut Microbiota by the Probiotic *Rummeliibacillus stabekisii* in Nile Tilapia (*Oreochromis niloticus*). Fish Shellfish Immunol..

[37] Saputra F., Shiu Y.-L., Chen Y.-C., Puspitasari A.W., Danata R.H., Liu C.-H., Hu S.-Y. (2016). Dietary Supplementation with Xylanase-Expressing *B. Amyloliquefaciens* R8 Improves Growth Performance and Enhances Immunity against *Aeromonas hydrophila* in Nile Tilapia (*Oreochromis niloticus*). Fish Shellfish Immunol..

[38] Zuo Z.-H., Shang B.-J., Shao Y.-C., Li W.-Y., Sun J.-S. (2019). Screening of Intestinal Probiotics and the Effects of Feeding Probiotics on the Growth, Immune, Digestive Enzyme Activity and Intestinal Flora of *Litopenaeus vannamei*. Fish Shellfish Immunol..

[39] Yi C.-C., Liu C.-H., Chuang K.-P., Chang Y.-T., Hu S.-Y. (2019). A Potential Probiotic *Chromobacterium aquaticum* with Bacteriocin-like Activity Enhances the Expression of Indicator Genes Associated with Nutrient Metabolism, Growth Performance and Innate Immunity against Pathogen Infections in Zebrafish (*Danio rerio*). Fish Shellfish Immunol..

[40] Lin Y.-S., Saputra F., Chen Y.-C., Hu S.-Y. (2019). Dietary Administration of Bacillus Amyloliquefaciens R8 Reduces Hepatic Oxidative Stress and Enhances Nutrient Metabolism and Immunity against *Aeromonas hydrophila* and *Streptococcus agalactiae* in Zebrafish (*Danio rerio*). Fish Shellfish Immunol..

[41] Hmani H., Daoud L., Jlidi M., Jalleli K., Ben Ali M., Hadj Brahim A., Bargui M., Dammak A., Ben Ali M. (2017). A *Bacillus subtilis* Strain as Probiotic in Poultry: Selection Based on in Vitro Functional Properties and Enzymatic Potentialities. J. Ind. Microbiol. Biotechnol..

[42] Midhun S.J., Neethu S., Vysakh A., Sunil M.A., Radhakrishnan E.K., Jyothis M. (2017). Antibacterial Activity of Autochthonous Bacteria Isolated from *Anabas testudineus* (Bloch, 1792) and It’s in Vitro Probiotic Characterization. Microb. Pathog..

[43] Olmos Soto J. (2017). Bacillus Probiotic Enzymes: External Auxiliary Apparatus to Avoid Digestive Deficiencies, Water Pollution, Diseases, and Economic Problems in Marine Cultivated Animals. Adv. Food Nutr. Res..

[44] Mohammadi G., Hafezieh M., Karimi A.A., Azra M.N., Van Doan H., Tapingkae W., Abdelrahman H.A., Dawood M.A.O. (2022). The Synergistic Effects of Plant Polysaccharide and *Pediococcus acidilactici* as a Synbiotic Additive on Growth, Antioxidant Status, Immune Response, and Resistance of Nile Tilapia (*Oreochromis niloticus*) against *Aeromonas hydrophila*. Fish Shellfish Immunol..

[45] Torres-Maravilla E., Parra M., Maisey K., Vargas R.A., Cabezas-Cruz A., Gonzalez A., Tello M., Bermúdez-Humarán L.G. (2024). Importance of Probiotics in Fish Aquaculture: Towards the Identification and Design of Novel Probiotics. Microorganisms.

[46] Yang S., Du J., Luo J., Zhou Y., Long Y., Xu G., Zhao L., Du Z., Yan T. (2019). Effects of Different Diets on the In-testinal Microbiota and Immunity of Common Carp (*Cyprinus carpio*). J. Appl. Microbiol..

[47] Setlow P. (2006). Spores of *Bacillus subtilis*: Their Resistance to and Killing by Radiation, Heat and Chemicals. J. Appl. Microbiol..

[48] Li X., Kong R., Wang J., Wu J., He K., Wang X. (2023). The Formation Mechanism of *Bacillus subtilis* Biofilm Surface Morphology under Competitive Environment. Can. J. Microbiol..

[49] Wang J., Li X., Kong R., Wu J., Wang X. (2022). Fractal Morphology Facilitates *Bacillus subtilis* Biofilm Growth. Environ. Sci. Pollut. Res..

[50] Wang Y., Tyler B.M., Wang Y. (2019). Defense and Counterdefense During Plant-Pathogenic Oomycete Infection. Annu. Rev. Microbiol..

[51] Mazanko M.S., Gorlov I.F., Prazdnova E.V., Makarenko M.S., Usatov A.V., Bren A.B., Chistyakov V.A., Tutelyan A.V., Komarova Z.B., Mosolova N.I. (2018). Bacillus Probiotic Supplementations Improve Laying Performance, Egg Quality, Hatching of Laying Hens, and Sperm Quality of Roosters. Probiotics Antimicrob. Proteins.

[52] Prazdnova E.V., Mazanko M.S., Chistyakov V.A., Denisenko Y.V., Makarenko M.S., Usatov A.V., Bren A.B., Tutelyan A.V., Komarova Z.B., Gorlov I.F. (2019). Effect of *Bacillus subtilis* KATMIRA1933 and *Bacillus amyloliquefaciens* B-1895 on the Productivity, Reproductive Aging, and Physiological Characteristics of Hens and Roosters. Benef. Microbes.

[53] Ponomareva E.N., Sorokina M.N., Grigoriev V.A., Mazanko M., Chistyakov V.A., Rudoy D.V. (2024). Probiotic *Bacillus amyloliquefaciens* B-1895 Improved Growth of Juvenile Trout. Food Sci. Anim. Resour..

[54] Soltani M., Pakzad K., Taheri-Mirghaed A., Mirzargar S., Shekarabi S.P.H., Yosefi P., Soleymani N. (2019). Dietary Application of the Probiotic *Lactobacillus plantarum* 426951 Enhances Immune Status and Growth of Rainbow Trout (*Oncorhynchus mykiss*) Vaccinated Against *Yersinia ruckeri*. Probiotics Antimicrob. Proteins.

[55] Oyugi D.O., Cucherousset J., Baker D.J., Britton J.R. (2012). Effects of Temperature on the Foraging and Growth Rate of Juvenile Common Carp, *Cyprinus carpio*. J. Therm. Biol..

[56] Heydarnejad M. (2012). Survival and Growth of Common Carp (*Cyprinus carpio* L.) Exposed to Different Water pH Levels. Turk. J. Vet. Anim. Sci..

[57] Ghodrati M., Hosseini Shekarabi S.P., Rajabi Islami H., Shenavar Masouleh A., Shamsaie Mehrgan M. (2021). Singular or Combined Dietary Administration of Multi-strain Probiotics and Multi-enzyme Influences Growth, Body Composition, Digestive Enzyme Activity, and Intestinal Morphology in Siberian Sturgeon (*Acipenser baerii*). Aquacult. Nutr..

[58] Pourgholam M.A., Khara H., Safari R., Sadati M.A.Y., Aramli M.S. (2016). Dietary Administration of *Lactobacillus plantarum* Enhanced Growth Performance and Innate Immune Response of Siberian Sturgeon, *Acipenser baerii*. Probiotics Antimicrob. Proteins.

[59] Gobi N., Vaseeharan B., Chen J.-C., Rekha R., Vijayakumar S., Anjugam M., Iswarya A. (2018). Dietary Supplementation of Probiotic *Bacillus licheniformis* Dahb1 Improves Growth Performance, Mucus and Serum Immune Parameters, Antioxidant Enzyme Activity as Well as Resistance against *Aeromonas hydrophila* in Tilapia *Oreochromis mossambicus*. Fish Shellfish Immunol..

[60] Geraylou Z., Souffreau C., Rurangwa E., De Meester L., Courtin C.M., Delcour J.A., Buyse J., Ollevier F. (2013). Effects of Dietary Arabinoxylan-Oligosaccharides (AXOS) and Endogenous Probiotics on the Growth Performance, Non-Specific Immunity and Gut Microbiota of Juvenile Siberian Sturgeon (*Acipenser baerii*). Fish Shellfish Immunol..

[61] Di J., Chu Z., Zhang S., Huang J., Du H., Wei Q. (2019). Evaluation of the Potential Probiotic *Bacillus subtilis* Isolated from Two Ancient Sturgeons on Growth Performance, Serum Immunity and Disease Resistance of *Acipenser dabryanus*. Fish Shellfish Immunol..

[62] Darafsh F., Soltani M., Abdolhay H.A., Shamsaei Mehrejan M. (2020). Improvement of Growth Performance, Digestive Enzymes and Body Composition of Persian Sturgeon (*Acipenser persicus*) Following Feeding on Probiotics: *Bacillus licheniformis*, *Bacillus subtilis* and *Saccharomyces cerevisiae*. Aquac. Res..

[63] Gautam V.A. (2022). DNA and RNA Isolation Techniques for Non-Experts. Techniques in Life Science and Biomedicine for the Non-Expert.

[64] Grant J.R., Enns E., Marinier E., Mandal A., Herman E.K., Chen C.-Y., Graham M., Van Domselaar G., Stothard P. (2023). Proksee: In-Depth Characterization and Visualization of Bacterial Genomes. Nucleic Acids Res..

[65] Schwengers O., Jelonek L., Dieckmann M.A., Beyvers S., Blom J., Goesmann A. (2021). Bakta: Rapid and Standardized Annotation of Bacterial Genomes via Alignment-Free Sequence Identification. Microb. Genom..

[66] Alcock B.P., Huynh W., Chalil R., Smith K.W., Raphenya A.R., Wlodarski M.A., Edalatmand A., Petkau A., Syed S.A., Tsang K.K. (2023). CARD 2023: Expanded Curation, Support for Machine Learning, and Resistome Prediction at the Comprehensive Antibiotic Resistance Database. Nucleic Acids Res..

[67] Kanehisa M., Sato Y., Morishima K. (2016). BlastKOALA and GhostKOALA: KEGG Tools for Functional Characterization of Genome and Metagenome Sequences. J. Mol. Biol..

[68] Blin K., Shaw S., Augustijn H.E., Reitz Z.L., Biermann F., Alanjary M., Fetter A., Terlouw B.R., Metcalf W.W., Helfrich E.J.N. (2023). antiSMASH 7.0: New and Improved Predictions for Detection, Regulation, Chemical Structures and Visualisation. Nucleic Acids Res..

[69] Chaumeil P.-A., Mussig A.J., Hugenholtz P., Parks D.H. (2022). GTDB-Tk v2: Memory Friendly Classification with the Genome Taxonomy Database. Bioinformatics.

[70] Parks D.H., Chuvochina M., Rinke C., Mussig A.J., Chaumeil P.-A., Hugenholtz P. (2022). GTDB: An Ongoing Census of Bacterial and Archaeal Diversity through a Phylogenetically Consistent, Rank Normalized and Complete Genome-Based Taxonomy. Nucleic Acids Res..

[71] Letunic I., Bork P. (2021). Interactive Tree Of Life (iTOL) v5: An Online Tool for Phylogenetic Tree Display and Annotation. Nucleic Acids Res..

[72] Prasetyo D., Zubaidah A., Cahya Putra R.D., Anne O., Ariansyah F. Growth Performance of Tilapia Fed Commercial Feed with Cellulolytic Bacteria from Ruminants. Proceedings of the BIO Web of Conferences: The 3rd and 4th International Conference on Bioenergy and Environmentally Sustainable Agriculture Technology (ICoN BEAT 2022 and 2023).

[73] Wang Y., Al Farraj D.A., Vijayaraghavan P., Hatamleh A.A., Biji G.D., Rady A.M. (2020). Host Associated Mixed Probiotic Bacteria Induced Digestive Enzymes in the Gut of Tiger Shrimp Penaeus Monodon. Saudi J. Biol. Sci..

[74] Gänzle M.G., Follador R. (2012). Metabolism of Oligosaccharides and Starch in Lactobacilli: A Review. Front. Microbiol..

[75] Blanco P., Hernando-Amado S., Reales-Calderon J.A., Corona F., Lira F., Alcalde-Rico M., Bernardini A., Sanchez M.B., Martinez J.L. (2016). Bacterial Multidrug Efflux Pumps: Much More Than Antibiotic Resistance Determinants. Microorganisms.

[76] Abdelsamad A.E.M., Said R.E.M., Assas M., Gaafar A.Y., Hamouda A.H., Mahdy A. (2024). Effects of Dietary Supplementation with *Bacillus velezensis* on the Growth Performance, Body Composition, Antioxidant, Immune-Related Gene Expression, and Histology of Pacific White Shrimp, *Litopenaeus vannamei*. BMC Vet. Res..

[77] Barale S.S., Ghane S.G., Sonawane K.D. (2022). Purification and Characterization of Antibacterial Surfactin Isoforms Produced by *Bacillus velezensis* SK. AMB Express.

[78] Jeon H.J., Song J.W., Lee C., Kim B., Park S.Y., Kim J.H., Han J.E., Park J.H. (2022). Antibacterial Activity of Bacillus Strains against Acute Hepatopancreatic Necrosis Disease-Causing *Vibrio campbellii* in Pacific White Leg Shrimp. Fishes.

[79] Sam-On M.F.S., Mustafa S., Mohd Hashim A., Yusof M.T., Zulkifly S., Malek A.Z.A., Roslan M.A.H., Mohd Asrore M.S. (2023). Mining the Genome of *Bacillus velezensis* FS26 for Probiotic Markers and Secondary Metabolites with Antimicrobial Properties against Aquaculture Pathogens. Microb. Pathog..

[80] Yang Q., Zhang H., You J., Yang J., Zhang Q., Zhao J., Aimaier R., Zhang J., Han S., Zhao H. (2022). Transcriptome and Metabolome Analyses Reveal That *Bacillus subtilis* BS-Z15 Lipopeptides Mycosubtilin Homologue Mediates Plant Defense Responses. Front. Plant Sci..

[81] Grady E.N., MacDonald J., Ho M.T., Weselowski B., McDowell T., Solomon O., Renaud J., Yuan Z.-C. (2019). Characterization and Complete Genome Analysis of the Surfactin-Producing, Plant-Protecting Bacterium *Bacillus velezensis* 9D-6. BMC Microbiol..

[82] Gu Q., Yang Y., Yuan Q., Shi G., Wu L., Lou Z., Huo R., Wu H., Borriss R., Gao X. (2017). Bacillomycin D Produced by *Bacillus amyloliquefaciens* Is Involved in the Antagonistic Interaction with the Plant-Pathogenic Fungus *Fusarium graminearum*. Appl. Environ. Microbiol..

[83] Vibe V., Kulikov M., Prazdnova E., Mazanko M., Chistyakov V., Rudoy D., Shevchenko V., Kulikova N. (2024). Effect of Growth Medium Composition on the Efficiency of Non-Ribosomal Synthesis in Bacteria of the Genus Bacillus. BIO Web Conf..

[84] Sword T.T., Abbas G.S.K., Bailey C.B. (2024). Cell-Free Protein Synthesis for Nonribosomal Peptide Synthetic Biology. Front. Nat. Prod..

[85] Yan H., Xin Z., Sang Z., Li X., Xie J., Wu J., Pang S., Wen Y., Wang W. (2025). A Rational Multi-Target Combination Strategy for Synergistic Improvement of Non-Ribosomal Peptide Production. Nat. Commun..

[86] Skripnichenko R.V., Chelombitskaya D.S., Prazdnova E.V., Kulikov M.P., Neurov A.M., Zaikina A.A., Grigoryev V.A., Sorokina M.N., Chistyakov V.A., Chikindas M.L. (2024). Potential Probiotic Bacillus Strains with Antioxidant and Antimutagenic Activity Increased Weight Gain and Altered Hsp70, Cxc, Tnfα, Il1β, and lysC Gene Expression in *Clarias gariepinus*. Fishes.

[87] Krysiak K., Konkol D., Korczyński M. (2021). Overview of the Use of Probiotics in Poultry Production. Animals.

[88] Barba-Vidal E., Martín-Orúe S.M., Castillejos L. (2019). Practical Aspects of the Use of Probiotics in Pig Production: A Review. Livest. Sci..

[89] Ma L., Wang L., Zhang Z., Xiao D. (2023). Research Progress of Biological Feed in Beef Cattle. Animals.

[90] Van Doan H., Hoseinifar S.H., Ringø E., Ángeles Esteban M., Dadar M., Dawood M.A., Faggio C. (2020). Host-associated probiotics: A key factor in sustainable aquaculture. Rev. Fish. Sci. Aquac..

[91] Zhang Z., Zhang H.L., Yang D.H., Hao Q., Yang H.W., Meng D.L., Zhou Z.G. (2024). *Lactobacillus rhamnosus* GG triggers intestinal epithelium injury in zebrafish revealing host dependent beneficial effects. IMeta.

[92] Rahayu S., Amoah K., Huang Y., Cai J., Wang B., Shija V.M., Jiang M. (2024). Probiotics application in aquaculture: Its potential effects, current status in China and future prospects. Front. Mar. Sci..

[93] Sugiura S.H. (2025). Evolutionary Loss of Acid-Secreting Stomach and Endoskeletal Ossification: A Phosphorus Perspective. Fishes.

[94] Radchikov V.F., Astrenkov A.V., Gadlevskaya N.N., Prodennia V.I., Djulić E.L., Rohalskaia S.U. (2022). Improvement of carp production efficiency through reduction of the cost of feed combinations. Mech. Electrif. Agric..

[95] Huang Z., Hou D., Zhou R., Zeng S., Xing C., Wei D., He J. (2021). Environmental water and sediment microbial communities shape intestine microbiota for host health: The central dogma in an anthropogenic aquaculture ecosystem. Front. Microbiol..

[96] Zhang B., Xiao J., Liu H., Zhai D., Wang Y., Liu S., Xia M. (2024). Vertical habitat preferences shape the fish gut microbiota in a shallow lake. Front. Microbiol..

[97] Khalid F., Khalid A., Fu Y., Hu Q., Zheng Y., Khan S., Wang Z. (2021). Potential of *Bacillus velezensis* as a Probiotic in Animal Feed: A Review. J. Microbiol..

[98] Wang X., Deng Z., Gao J. (2024). Exploring the Antibiotic Potential of Cultured ‘Unculturable’ Bacteria. Trends Microbiol..

[99] Li J., Wu Z.-B., Zhang Z., Zha J.-W., Qu S.-Y., Qi X.-Z., Wang G.-X., Ling F. (2019). Effects of Potential Probiotic *Bacillus velezensis* K2 on Growth, Immunity and Resistance to *Vibrio harveyi* Infection of Hybrid Grouper (*Epinephelus lanceolatus*♂ × *E. fuscoguttatus*♀). Fish Shellfish. Immunol..

[100] Liu X., Zeng S., Liu S., Wang G., Lai H., Zhao X., Bi S., Guo D., Chen X., Yi H. (2020). Identifying the Related Genes of Muscle Growth and Exploring the Functions by Compensatory Growth in Mandarin Fish (*Siniperca chuatsi*). Front. Physiol..

[101] Yin Y., Zhang Y., Hua Z., Wu A., Pan X., Yang J., Wang X. (2023). Muscle Transcriptome Analysis Provides New Insights into the Growth Gap between Fast- and Slow-Growing *Sinocyclocheilus grahami*. Front. Genet..

[102] Anee I.J., Alam S., Begum R.A., Shahjahan R.M., Khandaker A.M. (2021). The Role of Probiotics on Animal Health and Nutrition. J. Basic Appl. Zool..

[103] Jlidi M., Akremi I., Ibrahim A.H., Brabra W., Ali M.B., Ali M.B. (2022). Probiotic Properties of Bacillus Strains Isolated from the Gastrointestinal Tract against Pathogenic Vibriosis. Front. Mar. Sci..

[104] Wu P.-S., Liu C.-H., Hu S.-Y. (2021). Probiotic *Bacillus safensis* NPUST1 Administration Improves Growth Performance, Gut Microbiota, and Innate Immunity against *Streptococcus iniae* in Nile Tilapia (*Oreochromis niloticus*). Microorganisms.

[105] Assan D., Kuebutornye F.K.A., Hlordzi V., Chen H., Mraz J., Mustapha U.F., Abarike E.D. (2022). Effects of Probiotics on Digestive Enzymes of Fish (Finfish and Shellfish); Status and Prospects: A Mini Review. Comp. Biochem. Physiol. B Biochem. Mol. Biol..

[106] Kobayashi K. (2021). Diverse LXG Toxin and Antitoxin Systems Specifically Mediate Intraspecies Competition in *Bacillus subtilis* Biofilms. PLoS Genet.

[107] Yu T., Kong J., Zhang L., Gu X., Wang M., Guo T. (2019). New Crosstalk between Probiotics *Lactobacillus plantarum* and *Bacillus subtilis*. Sci. Rep..

[108] Payne J., Bellmer D., Jadeja R., Muriana P. (2024). The Potential of Bacillus Species as Probiotics in the Food Industry: A Review. Foods.

[109] Mazanko M.S., Prazdnova E.V., Kulikov M.P., Maltseva T.A., Rudoy D.V., Chikindas M.L. (2022). Antioxidant and Antimutagenic Properties of Probiotic Lactobacilli Determined Using LUX-Biosensors. Enzym. Microb. Technol..

[110] Pang X., Fu S.-J., Zhang Y.-G. (2016). Acclimation Temperature Alters the Relationship between Growth and Swimming Performance among Juvenile Common Carp (*Cyprinus carpio*). Comp. Biochem. Physiol. A Mol. Integr. Physiol..

[111] Yanbo W., Zirong X. (2006). Effect of Probiotics for Common Carp (*Cyprinus carpio*) Based on Growth Performance and Digestive Enzyme Activities. Anim. Feed. Sci. Technol..

[112] Ji Z., Lu X., Xue M., Fan Y., Tian J., Dong L., Zhu C., Wen H., Jiang M. (2023). The Probiotic Effects of Host-Associated *Bacillus velezensis* in Diets for Hybrid Yellow Catfish (*Pelteobagrus fulvidraco* ♀ × *Pelteo-bagrus vachelli* ♂). Anim. Nutr..

[113] Chen L., Lv C., Li B., Zhang H., Ren L., Zhang Q., Zhang X., Gao J., Sun C., Hu S. (2021). Effects of *Bacillus velezensis* Supplementation on the Growth Performance, Immune Responses, and Intestine Microbiota of *Litopenaeus vannamei*. Front. Mar. Sci..

[114] Zhang D.-X., Kang Y.-H., Zhan S., Zhao Z.-L., Jin S.-N., Chen C., Zhang L., Shen J.-Y., Wang C.-F., Wang G.-Q. (2019). Effect of *Bacillus velezensis* on *Aeromonas veronii*-Induced Intestinal Mucosal Barrier Function Damage and Inflammation in Crucian Carp (*Carassius auratus*). Front. Microbiol..

[115] Amoah K., Tan B., Zhang S., Chi S., Yang Q., Liu H., Yang Y., Zhang H., Dong X. (2023). Host Gut-Derived Bacillus Probiotics Supplementation Improves Growth Performance, Serum and Liver Immunity, Gut Health, and Resistive Capacity against *Vibrio harveyi* Infection in Hybrid Grouper (♀*Epinephelus fuscoguttatus* × ♂*Epinephelus lanceolatus*). Anim. Nutr..

[116] Ariyanto Y.S., Anika M. (2024). Probiotics on Commercial Fish Growth: A Meta-Analysis. J. Sumberd. Hayati.

[117] Chen X., Yi H., Liu S., Zhang Y., Su Y., Liu X., Bi S., Lai H., Zeng Z., Li G. (2021). Probiotics Improve Eating Disorders in Mandarin Fish (*Siniperca chuatsi*) Induced by a Pellet Feed Diet via Stimulating Immunity and Regulating Gut Microbiota. Microorganisms.

[118] Lee Y., Nguyen T.L., Roh H., Kim A., Park J., Lee J.-Y., Kang Y.-R., Kang H., Sohn M.-Y., Park C.-I. (2023). Mechanisms Underlying Probiotic Effects on Neurotransmission and Stress Resilience in Fish via Transcriptomic Profiling. Fish Shellfish Immunol..

[119] Zhang J., Huang M., Feng J., Chen Y., Li M., Meng X., Chang X. (2023). Effect of Beneficial Colonization of *Bacillus coagulans* NRS 609 on Growth Performance, Intestinal Health, Antioxidant Capacity, and Immune Response of Common Carp (*Cyprinus carpio* L.). Aquac. Nutr..

[120] Agrawal S., Acharya D., Adholeya A., Barrow C.J., Deshmukh S.K. (2017). Nonribosomal Peptides from Marine Microbes and Their Antimicrobial and Anticancer Potential. Front. Pharmacol..

[121] Hoste A.C.R., Smeralda W., Cugnet A., Brostaux Y., Deleu M., Garigliany M., Jacques P. (2024). The Structure of Lipopeptides Impacts Their Antiviral Activity and Mode of Action against SARS-CoV-2 in Vitro. Appl. Environ. Microbiol..

[122] Leistikow K.R., May D.S., Suh W.S., Vargas Asensio G., Schaenzer A.J., Currie C.R., Hristova K.R. (2024). *Bacillus subtilis*-Derived Peptides Disrupt Quorum Sensing and Biofilm Assembly in Multidrug-Resistant *Staphylococcus aureus*. mSystems.

[123] Horng Y.-B., Yu Y.-H., Dybus A., Hsiao F.S.-H., Cheng Y.-H. (2019). Antibacterial Activity of Bacillus Species-Derived Surfactin on *Brachyspira hyodysenteriae* and *Clostridium perfringens*. AMB Express.

[124] Wu L., Wu H., Chen L., Yu X., Borriss R., Gao X. (2015). Difficidin and Bacilysin from *Bacillus amyloliquefaciens* FZB42 Have Antibacterial Activity against *Xanthomonas oryzae* Rice Pathogens. Sci. Rep..

[125] Nannan C., Vu H.Q., Gillis A., Caulier S., Nguyen T.T.T., Mahillon J. (2021). Bacilysin within the *Bacillus subtilis* Group: Gene Prevalence versus Antagonistic Activity against Gram-Negative Foodborne Pathogens. J. Biotechnol..

[126] Li H., Han X., Dong Y., Xu S., Chen C., Feng Y., Cui Q., Li W. (2021). Bacillaenes: Decomposition Trigger Point and Biofilm Enhancement in Bacillus. ACS Omega.

[127] Chakraborty K., Kizhakkekalam V.K., Joy M., Dhara S. (2021). Difficidin Class of Polyketide Antibiotics from Marine Macroalga-Associated Bacillus as Promising Antibacterial Agents. Appl. Microbiol. Biotechnol..

[128] Yuan J., Zhao M., Li R., Huang Q., Rensing C., Raza W., Shen Q. (2016). Antibacterial Compounds-Macrolactin Alters the Soil Bacterial Community and Abundance of the Gene Encoding PKS. Front. Microbiol..

[129] Yu C., Liu X., Zhang X., Zhang M., Gu Y., Ali Q., Mohamed M.S.R., Xu J., Shi J., Gao X. (2021). Mycosubtilin Produced by *Bacillus subtilis* ATCC6633 Inhibits Growth and Mycotoxin Biosynthesis of *Fusarium graminearum* and *Fusarium verticillioides*. Toxins.

[130] Xu Z., Shao J., Li B., Yan X., Shen Q., Zhang R. (2013). Contribution of Bacillomycin D in *Bacillus amyloliquefaciens* SQR9 to Antifungal Activity and Biofilm Formation. Appl. Environ. Microbiol..

[131] Nam J., Alam S.T., Kang K., Choi J., Seo M.-H. (2021). Anti-Staphylococcal Activity of a Cyclic Lipopeptide, C15 -Bacillomycin D, Produced by *Bacillus velezensis* NST6. J. Appl. Microbiol..

[132] Lee D., Oh T., Kang B., Ahn J.S., Cho Y. (2022). Throughput Screening of *Bacillus subtilis* Strains That Abundantly Secrete Surfactin in Vitro Identifies Effective Probiotic Candidates. PLoS ONE.

[133] Neurov A.M., Zaikina A.A., Prazdnova E.V., Anuj R., Rudoy D.V. (2024). Modulation of Stress-Related Protein in the African Catfish (*Clarias gariepinus*) Using Bacillus-Based Non-Ribosomal Peptides. Microbiol. Res..

[134] Yang H., Wu J., Du H., Zhang H., Li J., Wei Q. (2022). Quantifying the Colonization of Environmental Microbes in the Fish Gut: A Case Study of Wild Fish Populations in the Yangtze River. Front. Microbiol..

[135] Chao R., Abel C., Malachi A.W., Nancy C., Bui C.T.D., Joseph C.N., Mark R.L. (2012). Identification of Bacillus strains for biological control of catfish pathogens. PLoS ONE.

[136] Giatsis C., Sipkema D., Ramiro-Garcia J., Bacanu G.M., Abernathy J., Verreth J., Verdegem M. (2016). Probiotic legacy effects on gut microbial assembly in tilapia larvae. Sci. Rep..

[137] Asaduzzaman M., Sofia E., Shakil A., Haque N.F., Khan M.N.A., Ikeda D., Kinoshita S., Abol-Munafi A.B. (2018). Host Gut-Derived Probiotic Bacteria Promote Hypertrophic Muscle Progression and Upregulate Growth-Related Gene Expression of Slow-Growing Malaysian Mahseer *Tor tambroides*. Aquac. Rep..

[138] Sadeghi J., Chaganti S.R., Heath D.D. (2023). Regulation of Host Gene Expression by Gastrointestinal Tract Microbiota in Chinook Salmon (*Oncorhynchus tshawytscha*). Mol. Ecol..

[139] Hemarajata P., Versalovic J. (2013). Effects of Probiotics on Gut Microbiota: Mechanisms of Intestinal Immunomodulation and Neuromodulation. Ther. Adv. Gastroenterol..

[140] Hines I.S., Santiago-Morales K.D., Ferguson C.S., Clarington J., Thompson M., Rauschenbach M., Levine U., Drahos D., Aylward F.O., Smith S.A. (2022). Steelhead Trout (*Oncorhynchus mykiss*) Fed Probiotic during the Earliest Developmental Stages Have Enhanced Growth Rates and Intestinal Microbiome Bacterial Diversity. Front. Mar. Sci..

[141] Ul Hassan H., Mohammad Ali Q., Ahmad N., Masood Z., Hossain Y., Gabol K., Khan W., Hussain M., Ali A., Attaullah M. (2021). Assessment of Growth Characteristics, the Survival Rate and Body Composition of Asian Sea Bass *Lates calcarifer* (Bloch, 1790) under Different Feeding Rates in Closed Aquaculture System. Saudi J. Biol. Sci..

[142] Shadrack R.S., Manabu I., Yokoyama S. (2021). Efficacy of Single and Mix Probiotic Bacteria Strain on Growth Indices, Physiological Condition and Bio-Chemical Composition of Juvenile Amberjack (*Seriola dumerili*). Aquac. Rep..

[143] Speare D.J., MacNair N., Hammell K.L. (1995). Demonstration of Tank Effect on Growth Indices of Juvenile Rainbow Trout (*Oncorhynchus mykiss*) during an Ad Libitum Feeding Trial. Am. J. Vet. Res..

[144] World Aquaculture Society. Meeting Abstract. Experimental Design and Statistical Power of Fish Growth Studies. https://www.was.org/MeetingAbstracts/ShowAbstract/42356.

[145] Rwezawula P., Waiswa Mwanja W., Vereecke N., Bossier P., Vanrompay D. (2025). Advancing Aquaculture Probiotic Discovery via an Innovative Protocol for Isolation of Indigenous, Heat and Salt Tolerant, Quorum Quenching Probiotic Candidates. Front. Microbiol..

[146] Zhao C., Men X., Dang Y., Zhou Y., Ren Y. (2023). Probiotics Mediate Intestinal Microbiome and Microbiota-Derived Metabolites Regulating the Growth and Immunity of Rainbow Trout (*Oncorhynchus mykiss*). Microbiol. Spectr..

[147] Serra C.R., Almeida E.M., Guerreiro I., Santos R., Merrifield D.L., Tavares F., Oliva-Teles A., Enes P. (2019). Selection of Carbohydrate-Active Probiotics from the Gut of Carnivorous Fish Fed Plant-Based Diets. Sci. Rep..

